# Enzymatic extraction improves intracellular protein recovery from the industrial carrageenan seaweed *Eucheuma denticulatum* revealed by quantitative, subcellular protein profiling: A high potential source of functional food ingredients

**DOI:** 10.1016/j.fochx.2021.100137

**Published:** 2021-10-20

**Authors:** Simon Gregersen, Anne-Sofie Havgaard Kongsted, Rikke Brønnum Nielsen, Søren Storck Hansen, Frederik Andersen Lau, Jacob Bisgaard Rasmussen, Susan Løvstad Holdt, Charlotte Jacobsen

**Affiliations:** a Department of Chemistry and Bioscience, Aalborg University, Denmark; b National Food Institute, Technical University of Denmark, Denmark

**Keywords:** Macroalgae, Enzymatic protein extraction, Proteomics, Bioinformatics, Process evaluation, Bioactive peptides

## Abstract

Seaweeds are regarded as a sustainable source of food protein, but protein extraction is severely impaired by the complex extracellular matrix. In this work, we investigated the protein-level effects of enzymatic extraction upstream of carrageenan extraction for the industrial red seaweed *Eucheuma denticulatum*. Combination of quantitative proteomics and bioinformatic prediction of subcellular localization was shown to have immense potential for process evaluation; even in the case of poorly annotated species such as *E. denticulatum.* Applying cell wall degrading enzymes markedly improved the relative recovery of intracellular proteins compared to treatment with proteolytic enzymes or no enzymatic treatment. Moreover, results suggest that proteomics data may prove useful for characterizing amino acid composition and that length-normalization is a viable approach for relative protein quantification in non-specific analysis. Importantly, the extracts were abundant in proteins, which contained both previously verified and novel, potential bioactive peptides, highlighting their potential for application as functional food ingredients.

## Highlights

• Enzymatic protein extracts from *Eucheuma denticulatum* were characterized by proteomics.
• Predicted subcellular localization was used to evaluate extraction efficiency.
• Extraction with cell wall degrading enzymes increased intracellular protein recovery.
• Proteomics analysis may provide an efficient way to estimate amino acid composition.
• Abundant proteins contain both validated and potential bioactive peptides for foods.

## Introduction

The demand for protein-rich foods is increasing worldwide, partly due to increasing world population and economic growth, enabling more people in poverty to eat more protein-rich foods (Godfray et al., 2018). This trend negatively impacts climate change, as production and processing of conventional animal-derived food protein generally emit high amounts of carbon dioxide equivalents compared to other sources of food protein (Nijdam, Rood, & Westhoek, 2012). Half of habitable land is used for agriculture, where around 80% is used for livestock farming and contributes to loss of biodiversity, geological problems, and increased risk of zoonotic disease emergence (Poore & Nemecek, 2018). These major problems related to conventional animal agriculture industry provide a strong case for investigating alternative sources of protein for human consumption. Amongst these, plant-based protein and food products are generally more accepted by consumers (Onwezen, Bouwman, Reinders, & Dagevos, 2021). In addition to their nutritional qualities, food protein may also be considered a highly valuable source of biologically active and/or functional peptides embedded in the parent protein (Sánchez & Vázquez, 2017). Bioactive peptides may be released by enzymatic proteolysis and may provide added value of the protein if they possess e.g. antioxidant or antimicrobial properties (Lorenzo et al., 2018). Moreover, enzymatic hydrolysis has also in been reported to improve the functional properties (e.g. emulsification) compared to the crude protein (Ashaolu, 2020). Additional factors, e.g. allergenicity and sensory properties of co-extracted phenolics, are relevant to consider when assessing the quality of a food protein source, however studies indicate that these aspects might, similarly to functional properties, be improved through e.g. enzymatic hydrolysis (Sha & Xiong, 2020).

In the plant protein category, seaweeds (although macroalgae are not by definition plants) are of particular interest and considered industrially relevant. Cultivation of seaweed does not take up agricultural land space and has no need for fresh water, but instead exploits mostly unutilized ocean space (Bleakley & Hayes, 2017). Furthermore, seaweeds are already farmed for hydrocolloids such as alginate, agar, and carrageenan, and thus represent an underutilized resource through industrial side streams (Bleakley and Hayes, 2017, Naseri et al., 2020). Moreover, some species of seaweed outcompete traditional plant protein sources in terms of productivity per unit area per year and several seaweeds contain similar amounts of total protein compared to traditional protein sources of both animal and plant origin, such as egg, meat, and soy (Bleakley & Hayes, 2017). A significant challenge in the application of seaweeds for food protein production is that total protein content varies significantly between species. Brown seaweeds generally have lower protein content (3–15% total protein DM basis), while green and particularly red seaweeds may have up to 47% protein (DM basis) (Biancarosa et al., 2016, Fleurence, 1999, Naseri et al., 2019). Moreover, protein content also depends significantly on seasonal variation, as has been show to vary as much as three-fold over a season for the red seaweed *Palmaria palmata* (Fleurence, 1999)*.*

Animal proteins generally contain high levels of essential amino acids (EAA), but come with high cholesterol and saturated fat levels if not processed (Hoffman & Falvo, 2004). Plant proteins are often deficient in one or more EAAs and are generally harder to digest compared to animal protein (Gorissen et al., 2018). Digestibility of seaweed protein varies between 51 and 95%, where brown seaweeds are usually harder to digest due to their high fiber- and phlorotannin content, while red seaweeds generally are more digestible (>70%), similar to other common plant-protein sources like fruit, legumes, grains and vegetables (69–92%) (Bleakley & Hayes, 2017). The specific amino acid (AA) composition is a key nutritional property of a food protein. The property is often evaluated by ratio between essential and non-essential AAs (ΣEAA/ΣNEAA). The study by Biancarose *et al*. (2016) presented amino acid profiles of an empirically representative sample of seaweeds showing that in terms of amino acid profiles, red seaweeds in general (ΣEAA/ΣNEAA = 0.63), have a slightly higher nutritional quality than green (ΣEAA/ΣNEAA = 0.61) and brown seaweeds (ΣEAA/ΣNEAA = 0.59).

The red seaweed *Euchema denticulatum* (commonly referred to by the trade name Spinosum) is primarily farmed for extraction of carrageenan for application in foods (Bixler & Porse, 2010). As such, side streams and/or residual biomass represent a highly underutilized resource of e.g. proteins for potential use in foods. Despite having a quite modest content (4–5%) of crude protein (Bixler & Porse, 2010), *E. denticulatum* displays a high dietary potential evaluated by the amino-acid profile (ΣEAA/ ΣNEAA = 0.862) (Naseri et al., 2019). Where the ΣEAA/ΣNEAA-ratios of seaweeds in general are similar to that of common plant protein sources, the ratio in *E. denticulatum* is actually higher than those of milk, beef and egg. Additionally, *E. denticulatum* was recently shown to be a source of proteins with embedded peptides displaying emulsifying (Yesiltas et al., 2021) and antioxidant (Submitted manuscript, Food Chemistry) properties, further highlighting the high potential for application in foods. In contrast to the conventional trial-and error approach, these peptides were identified by application of new bioinformatic prediction tools, which are emerging as strong, novel tools in the search for natural alternatives to replace chemical additives in foods.

A challenge in seaweed protein extraction is the disruption of the cell walls, as a large number of proteins are found intracellularly or embedded in the cell wall (Mæhre, Jensen, & Eilertsen, 2016). Moreover, the polysaccharide constituents of the cell wall create higher viscosity and ionic interactions with proteins, hindering extraction (Harnedy & FitzGerald, 2013). This was recently highlighted in a study, where application of hot water extraction at near-neutral pH resulted in a protein extract almost exclusively constituted by extracellular proteins (Gregersen et al., 2020). To overcome this and efficiently extract protein from seaweed, different methods have been employed (Bleakley & Hayes, 2017). Conventionally, physical processes, such as grinding or osmotic stress, paired with alkaline extraction at high temperatures have been employed for this purpose. The disadvantage of these methods consists in the difficulty to upscale in commercial production, due to costs and workload. A more scalable solution could be novel extraction methods such as ultrasound-assisted extraction, as it has the advantages of reducing downstream processing and simultaneously yielding a purer product (Kadam, Álvarez, Tiwari, & O’Donnell, 2017).

To ensure industrial relevance and sustainability, it is of particular interest to extract protein alongside carrageenan in a cost-effective manner and without impairing carrageenan yield or quality. In the hydrocolloid industry, the most common method of extracting carrageenan from *E. denticulatum* involves alkaline extraction at high pH (Bixler & Porse, 2010), thereby making step-wise extraction and isolation of protein and hydrocolloids problematic. The study by Naseri et al. (2020) investigated the possibility of multi-extraction of protein and carrageenan with the aim of maintaining the same yield and quality of carrageenan. Protein extraction was performed up-stream of carrageenan extraction, applying enzymatic pre-treatment in combination with *N*-acetyl-l-cysteine (NAC) assisted alkaline extraction, using the two enzymes Viscozyme® and Alcalase® separately, and the two enzymes Celluclast® and Shearzyme® in combination. Protein extraction efficiency was evaluated for each enzymatic treatment, and it was found that, based on Dumas-N protein content estimation, enzymatic extraction significantly improved protein recovery and that treatment with Alcalase® resulted in the highest extraction efficiency at 59.4% followed by treatment with Viscozyme® at 48.5% (Naseri et al., 2020). Furthermore, parameters for carrageenan yield and quality were also evaluated and showed that protein extraction using enzymatic pre-treatment had no negative impact on carrageenan, and for some parameters showed better quality than without protein extraction. A similar approach of enzymatic extraction has also been applied for *Palmaria palmata* with similar improvements of the extraction efficiency (Naseri, Marinho, Holdt, Bartela, & Jacobsen, 2020).

The application of mass spectrometry-based proteomics for in-depth studies of red seaweeds has also gained a significant amount of attention and has recently been thoroughly reviewed (Beaulieu, 2019). Such studies have mostly been related to cellular response to exogenous factors, covering aspects such as desiccation and stress tolerance (Wang et al., 2020), and pathogen response (Khan et al., 2018). Moreover, bottom-up proteomics has only found very limited application for studying the potential of seaweed proteins as a nutritional source through in depth characterization, but has rather been applied for identification of bioactive peptides in seaweed protein hydrolysates, obtained using the classical trial-and-error approach (Beaulieu, Bondu, Doiron, Rioux, & Turgeon, 2015). Using quantitative proteomics for evaluation of extraction methods and characterization of protein extracts for further bioinformatic analysis remains unchartered territory.

The aim of this study was to perform proteomic and bioinformatic characterization of the protein extracts from *E. denticulatum* obtained in Naseri et al. (2020) for evaluating their potential in food applications. Through such analysis, we aimed, for the first time, to evaluate how efficiently the enzymatic pre-treatments, in combination with NAC-assisted alkaline extraction, enables release of intracellular protein, as an explanation for improved protein recovery. Moreover, the applicability of using peptide- and protein-level quantitative data for estimating the AA composition in protein extracts was evaluated. Finally, identified and abundant proteins were investigated for content of previously verified bioactive peptides as well as potentially novel peptides with emulsifying and antioxidant properties using bioinformatic prediction.

## Materials and methods

Three enzymatic protein extracts from *E. denticulatum* were provided by The National Food Institute (Denmark) through the VALSEA (Valorization of red seaweed biomasses towards future sustainability) project, and obtained as previously described (Naseri et al., 2020). Briefly, the extracts were obtained by enzyme-assisted aqueous extraction at pH 7 with Viscozyme® (0.2% w/w), Alcalase® (0.2% w/w) or Shearzyme®+Celluclast® (0.2% w/w each) followed by NAC-assisted alkaline extraction (1 g/L NAC, 4 g/L NaOH). For simplicity, the extracts are referred to as V, A, and S + C, respectively. The alkaline extraction was performed three times and the supernatant was pooled with the liquid fractions from the enzymatic extraction. Protein precipitation was performed by adjusting pH to 3.5 with 2 M HCl and the pellet was subsequently freeze-dried and milled. Total protein was estimated by the Dumas method using an *N*-to-protein conversion factor of 5 and AA composition was determined by LC-MS analysis, as previously reported. All used chemicals were of HPLC-grade.

### Total soluble protein

The enzymatic protein extracts were dissolved in 100 mM NH_4_HCO_3_ (ABC, Sigma Aldrich, USA) with 0.2% sodium dodecyl sulphate (SDS, Applichem, Darmstadt, Germany) with pH 8.8 to reach a total protein concentration of 1 mg/mL based on Dumas-N (Naseri et al., 2020). Subsequently, the solubilized extracts were sonicated for 30 min using a Sonorex Digitec ultrasound bath (Bandelin, Berlin, Germany) and left overnight on a Stuart SRT6 roller mixer (Cole-Parmer, St. Neots, UK). The following day, remaining solids were precipitated by centrifugation at 4000 rpm for 15 min using a 5810R Tabletop centrifuge (Eppendorf, Hamburg, Germany) and the supernatant was collected. The solubilized extracts were concentrated by snap freezing in liquid nitrogen, incubated at −80 °C for 5–10 min and then freeze-dried using an Alpha 2–4 LSC plus freeze dryer (Martin Christ, Osterode am Harz, Germany). Samples were resuspended in 100 mM ABC with 0.2% SDS to reach concentration/volume reduction factors of 10 (extracts treated with Viscozyme® and Shearzyme®+Celluclast®) and 25 (extracts treated with Alcalase®). Total soluble protein was measured by Qubit protein assay (Thermo Scientific, Bremen, Germany) according to manufacturer guidelines.

### 1-D SDS-Page

For SDS-PAGE analysis, precast 4–20% Bis-Tris gradient gels (Genscript, Picastaway, USA) in a Tris-MOPS running buffer was used. The molecular weight marker was Pierce™ Unstained MW marker (Thermo Scientific). Maximum volume of 20 µL was loaded in the wells on the SDS-PAGE gel. Solubilized extracts were mixed 14:5:1 with a 4xSDS sample buffer (50 mM Tris (VWR, Leuven, Belgium) pH 6.8, 2% SDS, 10% glycerol (VWR), 0.02% bromophenol blue (Sigma-Aldrich), 12.4 mM ethylenediaminetetraacetic acid (EDTA, Carl Roth, Karlsruhe, Germany)) and 1 M dithiothreitol (DTT, Thermo Scientific), corresponding to a final DTT concentration of 50 mM. The samples incubated at 95 °C for 5 min to denature proteins. Running time for the gel was 45 min at 140 V. To visualize proteins, the gel was stained with Coomassie Brilliant Blue G250 (29 mM Coomassie Brilliant Blue G-250 (Sigma-Aldrich), 45% Ethanol (VWR), 10% Acetic acid (Fisher Scientific, Loughborough, UK)). Destaining of the gel was performed overnight with destain solution (8% Ethanol, 5% Acetic acid). Imaging of the gel was done using a ChemDoc MP Imaging System (Bio-Rad, Hercules, USA).

### In-gel digestion

In-gel digestion was done using the SDS-PAGE gradient gel with concentrated extracts, as previously described (García-Moreno et al., 2020). Briefly, the gel lanes were excised by scalpel and divided into four fractions: <14.4 kDa, 14.4–25 kDa, 25–45 kDa and > 45 kDa. Subsequently, each fraction was cut into pieces of 1x1 mm and washed by succeeding addition of 0.1 M ABC and 100% acetonitrile (ACN, VWR) until Coomassie was sufficiently removed. Proteins were reduced with DTT solution (10 mM DTT in 100 mM ABC) and Cys alkylation of proteins was done with iodacetamide (55 mM iodoacetamide (Fluka Biochemika, Buchs, Switzerland) in 100 mM ABC). Extracts were then dried using a Speedvac centrifuge (Thermo Scientific). Digestion was then performed with sequencing grade modified trypsin (Promega, Madison, USA) by covering gel pieces with a trypsin solution (12.5 ng/µL in 100 mM ABC). Following 15 min incubation on ice, the remaining trypsin solution was replaced with 100 mM ABC and incubated at 37 °C overnight. Peptides were extracted with successive addition of 0.5% formic acid (FA, Sigma Aldrich) and 100% ACN. Peptide extraction was repeated twice and extracts from their respective samples were pooled. The peptide extracts were dried down in a Speedvac centrifuge and suspended in 0.1% FA with 2% ACN.

### LC-MS/MS analysis

All samples were analyzed on an LC-ESI-MS/MS consisting of an EASY-nLC system (Thermo Scientific) coupled to a Q Exactive HF mass spectrometer (Thermo Scientific) with a Nanospray FLex ion source (Thermo Scientific). Peptides were loaded on a reverse phase Acclaim PEPMAP NANOTRAP column (C18, 100 Å, 100 μm. × 2 cm, (Thermo Scientific)) in solvent A (0.1% FA) followed by separation on a reverse phase ACCLAIM PEPMAP RSLC analytical column (C18, 100 Å, 75 um × 50 cm (Thermo Scientific)). Peptides were eluted by constant flow at 300 nL/min during a 60 min ramped gradient from 5 to 100% of solvent B (0.1% FA in 80% ACN, Fischer Scientific). MS was operated in positive ion and data dependent top-20 mode, were the (up to) 20 most intense MS1 precursors were selected for higher energy C-trap dissociation (HCD) fragmentation at 28 eV using a window of isolation of 1.2 *m*/*z*. Survey scans were obtained at a resolution of 60.000 at 200 *m*/*z* and HCD spectra were obtained at 15.000 at 200 *m*/*z*. Maximum ion injection time was set to 50 for MS and 45 for MS/MS scans. The underfill ratio was set to 3.5% and a dynamic exclusion of 30 *sec* was applied. During acquisition, “peptide match” and “exclude isotopes” were enabled.

### Quantitative analysis of LC-MS/MS data

MaxQuant v.1.6.10.43 (Cox & Mann, 2008) was used for identification and quantification of proteins as previously described (García-Moreno et al., 2020). Settings were maintained at default for tryptic analysis, using a *de-novo* transcriptome assembly of *E. denticulatum* (Gregersen et al., 2020) as protein reference database. Two missed cleavages and up to five modifications were allowed while a false discovery rate (FDR) of 1% was applied on both peptide and protein level. For quantification, both the iBAQ algorithm (Schwanhäusser et al., 2011) and the *I_L_^rel^* method (Gregersen et al., 2020) was applied following filtration of reverse hits and common contaminants. In addition to tryptic analysis, LC-MS/MS data was analyzed using both semi-specific and unspecific in silico digestion. Protein FDR was set at 5% while peptide FDR was maintained at 1%, as previously reported (Gregersen et al., 2020). For unspecific analysis, peptides of 5–65 AAs were included.

Following initial analysis and subsequent filtering of contaminants and reverse hits, data were subjected to quality-based filtering. This process largely resembles what was previously described (Gregersen et al., 2020), but also took into account correlation between technical replicates (i.e. protein identification in both replicates) and evaluated the impact on number of identified protein groups as well as relative, quantitative loss. Quantitative loss here refers to the relative abundance constituted by filtered proteins prior to the filtering step. A detailed description of quality parameters used in filtering and the threshold applied can be found in the Supporting Information. Venn diagrams, used for evaluating overlap of protein identifications between extracts and analysis methods, were plotted using Venny 2.1 (freely available at https://bioinfogp.cnb.csic.es/tools/venny/)

To evaluate correlation between replicates, analytical methods (tryptic, semi-specific, and unspecific digestion), as well as quantification methods (riBAQ and *I_L_^rel^*), the in-sample normalized relative abundances were plotted and the Pearson Correlation Coefficient (PCC) was computed in Perseus v.1.6.2.1 (Tyanova et al., 2016). PCC was, alongside quantitative loss, used to evaluate the impact on the different strategies for data filtering and clean-up.

### Protein homology and subcellular localization prediction

Due to lack of protein annotation, identification of abundant proteins was inferred using BLAST via UniProt KB/Swiss-Prot (Consortium et al., 2021). If no sufficient matches were identified, protein sequences were submitted to BLAST against the entire UniProtKB databse. The DeepLoc webtool (freely available at http://www.cbs.dtu.dk/services/DeepLoc/index.php) was applied to predict protein subcellular localization using BLOSUM 62 encoding (Almagro Armenteros, Sønderby, Sønderby, Nielsen, & Winther, 2017). Although a simplification, proteins from all non-extracellular compartments were pooled and represented as “intracellular”.

### Amino acid composition estimation

To estimate mean AA composition based on LC-MS/MS data, the approach of intensity-weighted, peptide-level AA frequency analysis was applied (Jafarpour et al., 2020). MS1 peptide intensities, following filtering of contaminants and reverse hits, were used as weight to determine the sample-level molar, AA frequency, $f_{\mathrm{AA}}^{\mathrm{peptide}}$, according to:

$$f_{\mathrm{AA}}^{\mathrm{peptide}}=\sum_{\mathrm{pep}=1}^{n}f_{\mathrm{AA}}^{p}*I_{\mathrm{rel}}^{p}$$

where $f_{\mathrm{AA}}^{p}$ is the relative frequency of a given AA in peptide *p* and $I_{\mathrm{rel}}^{p}$ is the relative MS1 intensity of peptide *p* (i.e. the intensity of *p* divided by the sum of intensities for all *n* peptides.

To compare the distribution to the quantitative AA analysis by LC-MS (mg/g extract) for the three extracts (Naseri et al., 2020), untreated *E. denticulatum* (Naseri et al., 2019), and to the minimum adult dietary requirements according to the World Health Organization/Food and Agricultural Organization of the United Nations/United Nations University (WHO/FAO/UNU, 2007), these data were converted to relative molar abundance. Initially, quantified AAs were converted into relative weight distribution by dividing the amount of the individual AA by the sum amount for all quantified AAs. The relative amount (by weight) of each AA was then divided by the molecular weight of the AA and multiplied by the weighted average AA molecular weight including water (128 g/mol).

In addition, AA composition was estimated using the length-normalized, relative protein quantification results (*I_L_^rel^*). For all identified proteins, the relative AA frequency (i.e. the number each AA occurs in a given protein sequence divided by its length) was computed using ProtrWeb (Xiao, Cao, Zhu, & Xu, 2015) (freely available as webtool at http://protr.org/). For sample-level AA composition, the relative molar frequency of each AA, $f_{\mathrm{AA}}^{\mathrm{protein}}$, was then calculated as:

$$f_{\mathrm{AA}}^{\mathrm{protein}}=\sum_{\mathrm{pro}=1}^{m}f_{\mathrm{AA}}^{q}*I_{\mathrm{rel}}^{q}$$

where $f_{\mathrm{AA}}^{q}$ is the relative frequency of a given AA in protein *q* and $I_{\mathrm{rel}}^{q}$ is the length-normalized intensity of protein q divided by the sum of length-normalized intensities for all m proteins (i.e. *I_L_^rel^*).

### Identification of verified and potential functional embedded peptides

Known and verified *E. denticulatum*-derived bioactive and functional peptides, were identified by cross-correlating identified proteins with previous studies on the topic (Yesiltas et al., 2021), (Submitted manuscript, Food Chemistry). Additionally, abundant proteins (*I_L_^rel^* > 2%) identified in this study, and not abundantly identified in our previous study (Gregersen et al., 2020), was submitted for prediction of embedded peptides with emulsifying and antioxidant properties using EmulsiPred (García-Moreno et al., 2020, García-Moreno et al., 2020) and AnOxPePred (Olsen et al., 2020), respectively. Clustering of predicted peptides based on sequence homology and identity (i.e. peptides from the regions in a protein) is performed as part of the AnOxPePred output file, while for EmulsiPred, the resulting data is represented as single peptides with uniquely associated scores. To simplify data representation, the top five scoring within each class of emulsifier (α, β, or γ), showing no sequence overlap with higher scoring peptide, were selected. Full lists of predicted peptides and their associated scores and parent proteins can be found in the Supplementary Data.

### Statistical analysis

Statistical analysis was performed in GraphPad Prism v.9.2.0 (ref) as one-way ANOVA using Tukey’s *post hoc* test at a 95% confidence level. For variables defined as sum of variables (e.g. the “intracellular” categorization), standard deviation for the sum variable was computed as the root of the sum of variances by considering the variables independent.

## Results and discussion

### Total soluble protein and 1D SDS-PAGE

Total soluble protein (TSP) was determined by Qubit for comparison with crude protein (by Dumas-N) and true protein (AA analysis by LC-MS) content previously described (Naseri et al., 2020). Although protein extraction efficiency was previously reported to be higher in extracts A (Naseri et al., 2020), these extracts contained the lowest protein content, while the protein content in extracts S + C was approximately double (Table 1.A). Protein contents determined as true protein (ΣAA) show no statistically significant difference (p < 0.05) compared to TSP by Qubit, when extracts are solubilized in detergent-containing buffer. The aqueous solubility of the extracts was markedly lower and displayed pH-dependency (data not shown), which should be kept in mind when considering potential applications. Crude protein, determined by Dumas-N with a conversion factor of 5.0, significantly (p > 0.05) overestimates protein content compared to true protein and TSP by Qubit. Dumas-N measures the amount of total nitrogen within a sample, including non-protein nitrogen and to account for this, a conversion factor is used. In a previous study of seaweeds, a *N*-to-protein conversion factor of 6.25 has been used widely used (Angell, Mata, de Nys, & Paul, 2016). Through *meta*-analysis, the conversion factor of 6.25 was shown to overestimate true protein content by up to 43% compared to true protein by ΣAA. Ultimately, a universal *N*-to-protein conversion factor of 5.0 was proposed for seaweeds. Nevertheless, a conversion factor of 5 with Dumas-N still appears to overestimate true protein content approximately 5–8 fold across the analyzed samples when comparing true protein by ΣAA (Naseri et al., 2020) or TSP by Qubit.

**Table 1:** Protein content (A), summary of correlation analysis, and quantitative loss evaluated from proteomics analysis by LC-MS/MS of the three (duplicate) enzymatic *E. denticulatum* extracts.

		Viscozyme (V)		Alcalase (A)		Shearzyme + Celluclast (S + C)	
		V-1	V-2	A-1	A-2	S + C-1	S + C-2
A. Protein content	Dumas-N × 5*	28.6 ± 0.44%^A^	20.7 ± 0.47%^B^	16.0 ± 0.10%^A^	13.2 ± 0.67%^B^	29.9 ± 1.00%^A^	23.5 ± 1.68%^B^
	ΣAA*	3.62 ± 0.29%^C^		2.55 ± 0.09%^C^		5.08 ± 0.23%^C^	
	Qubit	3.68 ± 0.83%^C^	3.09 ± 0.08%^C^	2.48 ± 0.04%^C^	2.28 ± 0.05%^C^	4.84 ± 0.09%^C^	3.58 ± 0.07%^C^

Initial quantification							
B. Replicate PCC	riBAQ	0.976		0.967		0.939	
	I_L_^rel^ (t)	0.966		0.959		0.874	
	I_L_^rel^ (s)	0.946		0.906		0.876	
	I_L_^rel^ (u)	0.960		0.919		0.846	
C. Method PCC	riBAQ vs. I_L_^rel^ (t)	0.986	0.986	0.991	0.986	0.961	0.988
	I_L_^rel^ (t) vs. I_L_^rel^ (s)	0.973	0.986	0.848	0.960	0.974	0.963
	I_L_^rel^ (t) vs. I_L_^rel^ (u)	0.940	0.974	0.775	0.845	0.753	0.934
	I_L_^rel^ (s) vs. I_L_^rel^ (u)	0.920	0.975	0.711	0.844	0.748	0.924
D. Method PCC (2reps avg)^1^	riBAQ vs. I_L_^rel^ (t)	0.986		0.988		0.979	
	I_L_^rel^ (t) vs. I_L_^rel^ (s)	0.985		0.922		0.969	
	I_L_^rel^ (t) vs. I_L_^rel^ (u)	0.965		0.823		0.862	
	I_L_^rel^ (s) vs. I_L_^rel^ (u)	0.955		0.776		0.853	
E. Quantitative loss (2reps avg)^2^	I_L_^rel^ (t)	3.8%	1.7%	2.5%	1.0%	1.3%	0.7%
	I_L_^rel^ (s)	3.3%	0.7%	2.9%	0.9%	1.4%	2.3%
	I_L_^rel^ (u)	5.2%	0.6%	4.4%	0.3%	0.7%	1.1%

Post-filtering re-quantification							
F. Method PCC (2reps avg)^1^	I_L_^rel^ (t) vs. I_L_^rel^ (s)	0.984		0.919		0.972	
	I_L_^rel^ (t) vs. I_L_^rel^ (u)	0.970		0.837		0.866	
	I_L_^rel^ (s) vs. I_L_^rel^ (u)	0.960		0.786		0.854	
G. Quantitative loss	I_L_^rel^ (t)	1.7%	0.4%	0.7%	0.8%	3.3%	5.9%
	I_L_^rel^ (s)	2.2%	2.5%	0.6%	1.3%	3.0%	4.6%
	I_L_^rel^ (u)	8.4%	3.7%	4.0%	11.9%	6.3%	4.9%
H. Quantitative loss (2reps avg)^2^	I_L_^rel^ (t)	4.3%	2.1%	2.6%	1.3%	3.3%	6.1%
	I_L_^rel^ (s)	4.1%	3.2%	3.1%	1.6%	3.0%	5.7%
	I_L_^rel^ (u)	10.0%	4.7%	8.2%	12.3%	6.6%	5.4%

Suspect outliers removed							
I. Method PCC (2reps avg)^1^	I_L_^rel^ (t) vs. I_L_^rel^ (s)	0.997		0.983		0.994	
	I_L_^rel^ (t) vs. I_L_^rel^ (u)	0.995		0.984		0.990	
	I_L_^rel^ (s) vs. I_L_^rel^ (u)	0.996		0.991		0.996	
J. Quantitative loss (2reps avg)^2^	I_L_^rel^ (t)	4.3%	2.1%	2.6%	1.3%	3.3%	6.1%
	I_L_^rel^ (s)	10.6%	6.8%	24.1%	6.6%	7.6%	12.9%
	I_L_^rel^ (u)	18.8%	9.5%	31.4%	24.7%	25.2%	16.0%
K. Between extract PCC^3^	Extract V	–		0.880		0.898	
	Extract A	–		–		0.849	

*Protein content by Dumas-N and ΣAA in accordance with (Naseri et al., 2020).

1 Duplicate average with identification in both technical replicates required.

2 Loss of total relative abundance (compared to initial quantification) be requiring identification in both technical replicates.

3 Duplicate average with identification in both technical replicates required based on *I_L_^rel^* from semi-specific analysis.

A, B, and ^C^ indicate grouped measurements (within the same extraction method but across replicates) with no statistically difference.

For the initial quantification, correlation of relative quantification is evaluated by the Pearson Correlation Coefficient (PCC) and listed for between replicate PCC (B) and between method PCC (C + D) as well as the quantitative loss by requiring identification in both technical replicates (E). Following quality-based filtering of protein identifications, correlation is evaluated by the between method PCC (F) and the quantitative loss by requiring protein identification in one (G) or both (H) technical replicates. Finally, following manual exclusion of suspect outliers, the correlation is evaluated by between-method PCC (I) and quantitative loss requiring identification in both technical replicates (J) in addition to between-extract PCC by I_L_^rel^ using semi-specific analysis (K). Scatter plots used for determination of PCCs can be found in the supplementary information.

In addition to the low protein content observed in the solubilized extracts, only a few and very vague bands were visible by SDS-PAGE analysis (Figure S1). The bands were observed around 18 kDa, 25 kDa, and 28 kDa. Moreover, smears were observed along the gel for all samples. The smears were mostly concentrated towards the lower MW regions and extracts A displayed slightly more intense smears while the smears also extended further down in the MW range compared to V and S + C extracts. As Alcalase is an endoprotease, (partial) protein hydrolysis during extraction would explain the higher content of, presumably, smaller peptides in the A extracts. Co-extracted cellular constituents such as DNA, lipids, and carbohydrates as well as significantly modified proteins (e.g. glycoproteins) are likely causes to smears observed in SDS-PAGE analysis (Gregersen et al., 2020). Observed smears are comparable to the appearance of *E. denticulatum* extracts obtained through hot water extraction at near neutral pH (Gregersen et al., 2020), but by no means comparable to extracts obtained using optimized conditions (Rosni et al., 2015). This procedure includes e.g. phenolic treatment and is therefore incompatible with scalable and cost-effective production of food-grade protein.

### Quantitative LC-MS/MS and data quality assessment

**Fig. 1:**
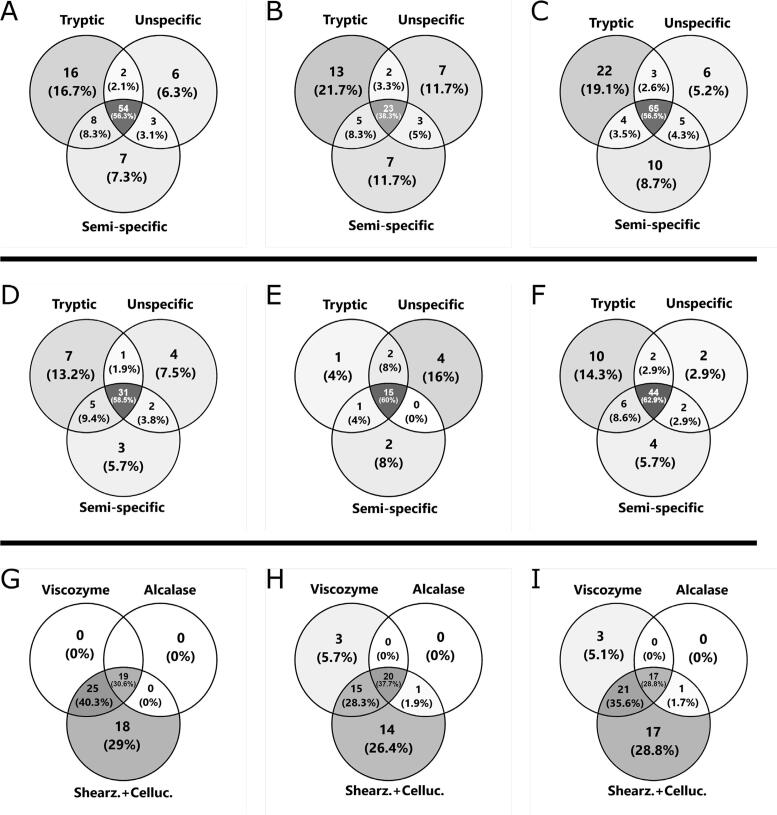
Venn diagrams showing the overlap of identified protein groups. *Top:* Initial identification and quantification showing overlap between analysis methods for extracts V (A), A (B) and S + C (C). *Middle*: Overlap of identified protein groups between analysis methods for extracts V (D), A (E) and S + C (F) following quality-based filtering. *Bottom*: Overlap of identified protein groups between enzymatic extracts for tryptic (G), unspecific (H), and semi-specific (I) analysis.

Following initial analysis of the three duplicate extracts by tryptic, semi-specific, and unspecific analysis, a total of 134 protein groups were identified (after merging overlapping protein groups between analytical methods). The highest number of protein groups were identified in the S + C extracts while the lowest number of protein identification were obtained for extracts A (Table S1). However, a large proportion of identified protein groups (24–51% across extract duplicates and analytical method) were only found in one replicate, indicating that the procedure for extraction may have issues regarding protein level reproducibility. Nevertheless, the vast majority of the protein groups identified in just one of the duplicates are of very low abundance. Requiring duplicate protein group identification only resulted in a quantitative loss of 0.6–5.2% across extract replicates and analytical methods (Table 1.E), illustrating that the abundant proteins are indeed identified in both technical replicates. As seen from Table 1.B, correlation between replicates varied between extracts and analysis method (PCC 0.85–0.98), where the best reproducibility was generally seen in extract V (Figure S2). This is also reflected in the cross-method correlation (Table 1.C), where extracts V displayed quite good correlation (PCC > 0.92), while unspecific analysis of particularly extracts A and S + C-1 resulted in much worse correlation (PCC 0.71–0.85). This may also be a direct consequence of inconsistent protein identifications across analytical methods (Fig. 1, A-C) for extract A (lowest degree of shared protein identifications, 38%) compared to the two other extracts (∼56% shared identifications between all three methods). Nevertheless, it was noteworthy that there is very high cross-method correlation between riBAQ and tryptic I_L_^rel^ (PCC > 0.96), highlighting that length-normalized relative quantification is in fact a viable alternative to iBAQ, in agreement with previous findings (Gregersen et al., 2020, Wiśniewski, 2017). Such good correlation is also found between I_L_^rel^ using both tryptic and semi-tryptic analysis in all extracts apart from A-1.

Requiring protein identification in both replicates for each extract (Table 1.D), improves correlations of poorly correlated extracts but at the cost of decreased correlation for better correlating extracts across analytical methods (Figure S3). However, requiring duplicate identification drastically increased the degree of shared proteins to 60% for extract A across methods (Fig. 1, D-F). As the main objective of this study was unambiguous identification of abundant proteins, and because 92 of the 134 identified protein groups had an Andromeda protein score below 40 (see Supplementary Data), extensive evaluation and filtering of protein identifications was deemed necessary before a reliable quantification could be achieved.

Following a quality-based filtering (see Supplementary Information for detailed description) overall adhering to a previously described process (Gregersen et al., 2020), the number of identified protein groups was reduced to 74 across replicates and analytical methods (Table S1). This resulted in a quite varied quantitative loss (Table 1.G + H) which was not particularly dependent on the requirement of duplicate identification (1.3–12.3%) or not (0.4–11.9%), but had a more pronounced impact on unspecific analysis. This may be related to the architecture of the Andromeda scoring algorithm, as the size of the decoy database for peptide spectrum matching (PSM) by definition is much larger in unspecific analysis compared to specific digestion, thereby increasing the probability for a lower Andromeda score for unspecific PSMs (Swaney, Wenger, & Coon, 2010). Nevertheless, the significant reduction in protein groups and resulting quantitative loss had only very little effect on the cross-method correlation, as seen in Table 1.F (Figure S4). Although the number of duplicate identifications between extract replicates is slightly decreased, the relative share of identifications in just one replicate is reduced to a higher extent (Table S1), which also adds to the improved correlation between extract replicate quantification (Table 1). Ultimately, a list of 19 protein groups, which were determined to be of high abundance (>2%) in any of the (averaged) extracts across analytical methods, was obtained (Table 2). From this, it was evident that some very high abundance proteins were exclusive for either a specific extract or a specific analysis method. This led us to further investigate the protein identifications, which were responsible for the poor cross-method correlation.

**Table 2:** Overview of highly abundant proteins (>2%) identified across extracts and analysis methods following quality-based filtering and subsequent re-quantification. Proteins are listed by ID according to the *de novo* reference database^1^ and their relative abundances are listed as duplicate mean across enzymatic extracts and analysis methods. For each protein, the results of BLAST^2^ analysis are listed by target AC# and BLAST score and the predicted subcellular localization^3^ is indicated.

		Viscozyme				Alcalase				Shearzyme + Celluclast						
Protein ID^1^	MW [kDa]	riBAQ	I_L_^rel^ (t)	**I_L_^rel^ (s)**	I_L_^rel^ (u)	riBAQ	I_L_^rel^ (t)	**I_L_^rel^ (s)**	I_L_^rel^ (u)	riBAQ	I_L_^rel^ (t)	**I_L_^rel^ (s)**	I_L_^rel^ (u)	BLAST Result^2^		Subcellular localization^3^
														Target AC#	Score	
c10861_g1_i1	27.5	0.8%	0.6%	**0.5%**	0.5%	7.1%	5.9%	**3.7%**	2.8%	0.8%	0.6%	**0.6%**	0.4%	Q95LI2	83	Extracellular
c13533_g1_i1	15.3	3.4%	2.7%	**2.2%**	2.7%	0.0%	0.0%	**0.0%**	0.0%	5.3%	4.1%	**4.3%**	4.2%	Q7VH33	66	Extracellular
c13559_g1_i1 c6825_g1_i1	13.5	1.6%	2.9%	**2.2%**	0.5%	0.3%	0.5%	**0.0%**	0.2%	4.8%	8.1%	**8.1%**	6.6%	Q64475	432	Cytoplasm
c14987_g1_i1	43.1	0.0%	0.0%	**0.0%**	0.2%	0.0%	0.0%	**0.0%**	2.2%	0.0%	0.0%	**0.0%**	0.2%	Q93Y35	900	Cytoplasm
c1505_g2_i1	15.8	33.8%	26.9%	**27.4%**	27.7%	38.8%	32.7%	**30.8%**	29.2%	31.7%	24.5%	**26.4%**	26.2%	C8V7C6	84	Extracellular
c1545_g1_i1	11.5	2.3%	3.0%	**2.8%**	3.2%	0.0%	0.0%	**0.0%**	0.0%	6.2%	7.8%	**7.4%**	7.1%	Q8T7J8	499	Nucleus
c17304_g1_i1	28.0	8.0%	9.9%	**9.8%**	8.7%	3.3%	4.4%	**3.9%**	2.6%	2.5%	3.0%	**2.3%**	2.3%	P84331	1393	Extracellular
c17933_g1_i1	14.6	0.0%	0.0%	**5.2%**	0.0%	0.0%	0.0%	**13.5%**	0.0%	0.0%	0.0%	**6.2%**	0.0%	Q8LPN7	76	Endoplasmic reticulum
c24_g1_i1	18.9	0.0%	0.0%	**0.0%**	0.0%	0.0%	0.0%	**3.4%**	0.0%	0.0%	0.0%	**0.2%**	0.0%	Q66628	82	Mitochondrion
c4419_g1_i1	14.4	1.2%	1.7%	**1.7%**	1.7%	0.0%	0.0%	**0.0%**	0.0%	1.8%	2.4%	**2.0%**	1.0%	P16569	197	Plastid
c6313_g1_i1	21.2	1.2%	1.4%	**1.3%**	1.4%	5.1%	6.2%	**5.7%**	5.8%	0.9%	1.0%	**1.1%**	1.0%			Extracellular
c6405_g1_i2	11.1	0.0%	0.0%	**0.0%**	7.6%	0.0%	0.0%	**0.0%**	20.6%	0.0%	0.0%	**0.0%**	15.5%	Q8TGK9	183	Plastid
c6656_g1_i1	43.0	2.2%	2.5%	**1.9%**	2.0%	0.1%	0.2%	**0.1%**	0.1%	2.1%	2.3%	**1.8%**	1.5%	Q944G9	1086	Plastid
c6963_g1_i1	22.0	2.4%	2.6%	**2.7%**	2.7%	0.9%	1.0%	**0.9%**	0.9%	4.9%	5.1%	**5.2%**	5.5%	P15214	320	Cytoplasm
c7052_g1_i1	24.2	8.3%	9.8%	**8.6%**	9.5%	14.8%	18.4%	**14.6%**	12.6%	5.8%	6.6%	**6.9%**	5.2%	P0A3U9	303	Extracellular
c7052_g1_i2	24.0	3.6%	3.6%	**4.3%**	4.5%	11.1%	11.5%	**9.9%**	10.3%	2.7%	2.5%	**2.7%**	2.6%	P0A3U9	331	Extracellular
c7216_g1_i1	25.4	3.0%	2.9%	**2.5%**	2.2%	11.3%	11.6%	**5.4%**	5.9%	5.3%	4.9%	**2.8%**	2.8%	Q9W770	167	Extracellular
c7502_g1_i1	13.4	0.0%	0.0%	**0.0%**	0.0%	0.0%	0.0%	**0.0%**	0.0%	2.2%	2.3%	**1.4%**	1.4%	Q6CK59	492	Cytoplasm
c907_g1_i1	14.8	15.7%	16.2%	**15.1%**	16.2%	5.1%	5.6%	**5.3%**	5.0%	7.0%	7.2%	**6.6%**	6.8%	P0CH07	566	Nucleus

1 Protein ID in accordance with (Gregersen et al., 2020).

2 BLAST results were obtained against UniProtKB (Consortium et al., 2021).

3 Subcellular localization as predicted by DeepLoc (Almagro Armenteros et al., 2017).

In the manual data curation, particularly two identified protein groups, which were not excluded by quality-based filtering, were found to be responsible for low cross-method correlation. One of these, c17933_g1_i1, was identified only through semi-specific analysis but identified in all extract replicates. It was furthermore only identified by a single peptide (SLFSLLR), which maps to>5000 protein entries in Uniprot (Consortium et al., 2021), and the Andromeda protein score was very low (6.5). Similarly, c6405_g1_i2 was found exclusively by unspecific analysis but in all extract replicates and only identified by a single peptide (LSFLSRL), which maps to>3000 proteins in Uniprot. Non-specific peptide identification was expected for particularly extracts A, given that Alcalase is an endoprotease. And although non-specific proteolytic activity has been reported for trypsin under sub-optimal conditions (Fang et al., 2015), the identification and comparable MS1 peptide intensities across all extract replicates, makes it unlikely to be the cause for this observation. As protein identification also was based on only one PSM, these identifications were regarded questionable and may, if the PSM was indeed correct, rather be associated with exogenous protein contamination. Removing just these two suspect outliers (i.e. potential false positives) followed by re-quantification improved cross-method correlation (Table 1.I) significantly to > 0.98 (Figure S5), but at the cost of a significant quantitative loss (Table 1.J) for especially unspecific analysis.

As *E. denticulatum* is quite poorly characterized and annotated, BLAST analysis was performed to gain further insight into the potential nature and function of the identified, abundant proteins (Table 2). From this analysis, several high scoring BLAST targets with very high sequence identity (Table S2) were identified. Among these, c17304_g1_i1 showed > 95% identity with Lectin ESA-2 from *Eucheuma serra*. This presumed lectin was found in very high abundance in extracts V (8.0–9.9% across analysis methods) and identified with high confidence based on sequence coverage (31%) and the maximum Andromeda score of 323 (see Supplementary Data). Lectins isolated from *E. denticulatum* have previously been shown to inhibit growth of pathogenic bacteria (Hung, Hirayama, Ly, & Hori, 2015). Furthermore, mannose-specific algal lectins have shown potential as both a virucidal agent against HIV-I infection and as cancer biomarkers and anti-cancer drugs (Barre, Simplicien, Benoist, Damme, & Rougé, 2019). Nevertheless, lectins are often regarded as anti-nutritional and have been reported to reduce digestibility and biological value of food proteins (Ramos, Mota, Teixeira, Cavada, & Moreira, 1998). As such, further processing of the protein extracts may be needed for food application. Identification of lectin also fits well with vague band at ∼ 28 kDa observed in SDS-PAGE analysis (Figure S1). Among other high identity BLAST matches, expected proteins such as histones (c13559_g1_i1; c6825_g1_i1; c1545_g1_i1; c7502_g1_i1) and ribosomal subunits (c907_g1_i1) are found. As these types of proteins are highly conserved across species, it cannot be excluded that the abundance of these proteins could potentially be overestimated as a result of exogenous contaminant proteins. The, by far, most abundant protein identified across all extracts and analysis methods was c1505_g2_i1 (24.5–38.8%). This protein was similarly found in very high abundance in hot-water *E. denticulatum* extracts (Gregersen et al., 2020), but the nature and function of this protein remains unknown. Similarly to the hot-water extracts, two isoforms of the same protein (c7052_g1_i1 and c7052_g1_i2) were also identified in high abundance in all extracts (7.7–30.0%), and in particularly high abundance in extracts A (>22.9%), while the third highly abundant protein in the hot-water extract, c6313_g1_i1 (32%), was found in a much lower abundance in the enzymatic extracts (0.9–6.2%) with the highest abundance in the A extracts (>5.1%).

### Evaluation of enzymatic extraction by subcellular protein distribution

**Fig. 2:**
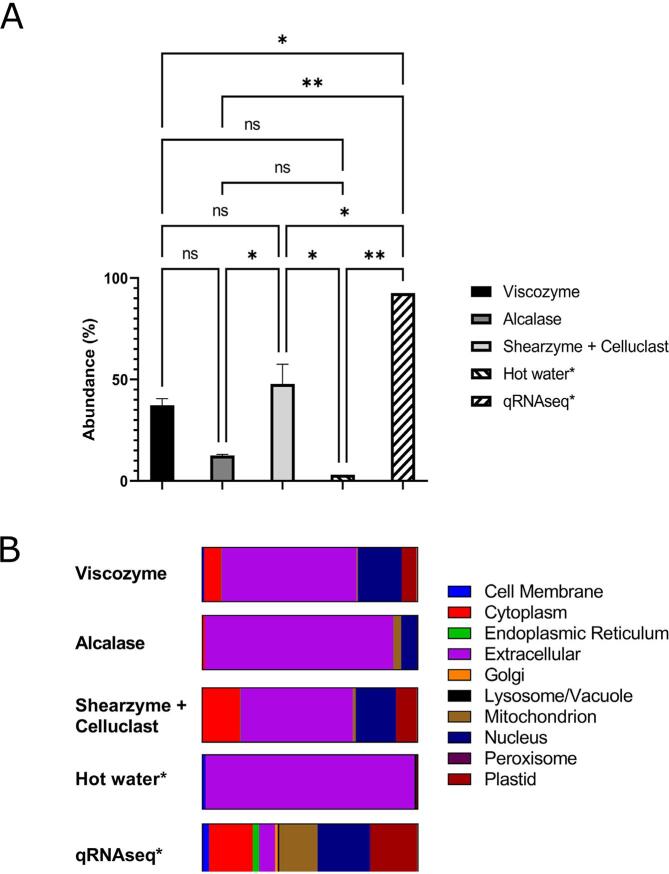
Distribution of quantified proteins according to predicted subcellular localization evaluated by semi-specific I_L_^rel^. A: Relative proportion of proteins allocated to the binned compartment “intracellular” (i.e. non-extracellular) by extraction method. Statistically difference between means evaluated by Tukey’s *post hoc* test in one-way ANOVA analysis is indicated by *(p < 0.05) and **(p < 0.01), while not significant is denoted by “ns”. B: Relative proportion of proteins allocated to each subcellular compartment as predicted by DeepLoc, presented as a stacked bar plot for each extraction method. *According to (Gregersen et al., 2020).

Subcellular distribution of proteins in the extracts was investigated by sequence analysis and localization prediction using DeepLoc (Almagro Armenteros et al., 2017). Evaluating the efficiency of enzymatic extraction method for liberating protein from within the plant cells was possible using simple binning of all non-extracellular proteins (termed “intracellular” for simplicity). As seen from Figure S6A, the distribution differs between extracts, and the relative proportion of extracellular protein appeared much higher for the A extracts. However, due to the quite high variance between only two technical replicates, the differences were not statistically significant (p > 0.05) between replicate means. By removing the two suspect outliers described above (which are both “intracellular” and c17933_g1_i1 being highly abundant in particularly extracts A as well as exclusive for semi-specific analysis), significant differences between means were seen despite the low number of replicates (Fig. 2A). For the abundance of intracellular proteins, the hot water extract of *E. denticulatum* was only statistically different (p < 0.05) from the S + C enzymatic extracts, while qRNAseq (by rTPM according to (Gregersen et al., 2020)), was significantly higher (p < 0.05) than all other extracts. Between enzymatic extracts, only A and S + C were significantly different, although there were notable differences between extracts A and V as well. Examining the subcellular compartmental distribution (Fig. 2B), extracts V and S + C had markedly higher content of cytoplasmic, nuclear and plastid proteins compared to extract A which, in turn, resembled the hot water extract to a much higher degree. This was even clearer following removal of the suspect outliers (Figure S6B). Moreover, removing the two suspect outliers also made the subcellular distribution much more comparable between analysis methods (Figure S7A) compared to before (Figure S7B). This underlines the applicability of length-normalized relative intensity using semi-specific analysis, I_L_^rel^ (s), if sufficient data curation and validation is employed to ensure high confidence protein quantification prior to quantification.

Applying cell wall degrading enzymes for extraction did indeed result in markedly higher (and in the case of S + C also statistically significant) release and recovery of intracellular proteins compared to the application of a proteolytic enzymes such as Alcalase® or no enzymatic treatment. Moreover, the relative abundance ascribed to non-extracellular proteins seemed to follow the same trend as seen for the content of TSP. Although this was not in agreement with the extraction efficiency, evaluated by Dumas-N previously described (Naseri et al., 2020), it may be reasonable to assume that increasing leakage of intracellular proteins by application of cell wall degrading enzymes ultimately will result in increased recovery of total protein. As these findings were also in line with true protein determination by ΣAA (Naseri et al., 2020), it further highlights that protein determination by Dumas may not always be a viable approach. Ultimately, the combination of TSP, length-normalized quantitative bottom-up proteomics, and bioinformatic analysis indicates that the combination of Shearzyme® and Celluclast® seemed to produce the extract with highest protein content as a direct consequence of more efficient cell lysis and increased release of intracellular proteins.

### Amino acid composition by shotgun proteomics

Conventionally, determination of amino acid (AA) composition in a protein extract is performed by full protein hydrolysis using heat and hydrochloric acid followed by quantification of individual AAs by LC-MS. Although this approach for AA analysis (commonly referred to simply as AAA) is robust and widely applied, there are severe limitations and drawbacks for a complete characterization of the AA profile of proteins (Rutherfurd & Gilani, 2009). During acid hydrolysis, Asn and Gln are deamidated and converted into Asp and Glu, respectively, thereby making it impossible to distinguish between the amine and carboxylic acid forms of Asx and Glx. Hydroxyl-containing AAs (Thr and Ser) are partially destroyed and losses of up to 15% may be encountered. Sulphur-containing AAs (Cys and Met) may be destroyed or oxidized during hydrolysis, and often, Cys is only quantified in the disulfide cystine form. Tyr may be halogenated during hydrolysis, thereby reducing quantitative recovery, while the hydrolysis efficiency for highly hydrophobic AAs (Val and Ile) is reduced, also resulting in lower recoveries. Finally, Trp may be completely destroyed during acid hydrolysis, resulting in decreased or even no quantitative recovery. Although numerous attempts have been made to resolve these challenges (Rutherfurd & Gilani, 2009), they all require significant sample work-up and chemical modifications prior to analysis. Furthermore, one method may facilitate quantitative recovery for a specific AA, but may concurrently impair the quantification of others. Ultimately, no universal method exists, which can provide a full AA profile through one-shot analysis. Based on the depth and quantitative potential of proteomics by LC-MS/MS, AA composition may be an added layer of extractable information from such analysis.

**Fig. 3:**
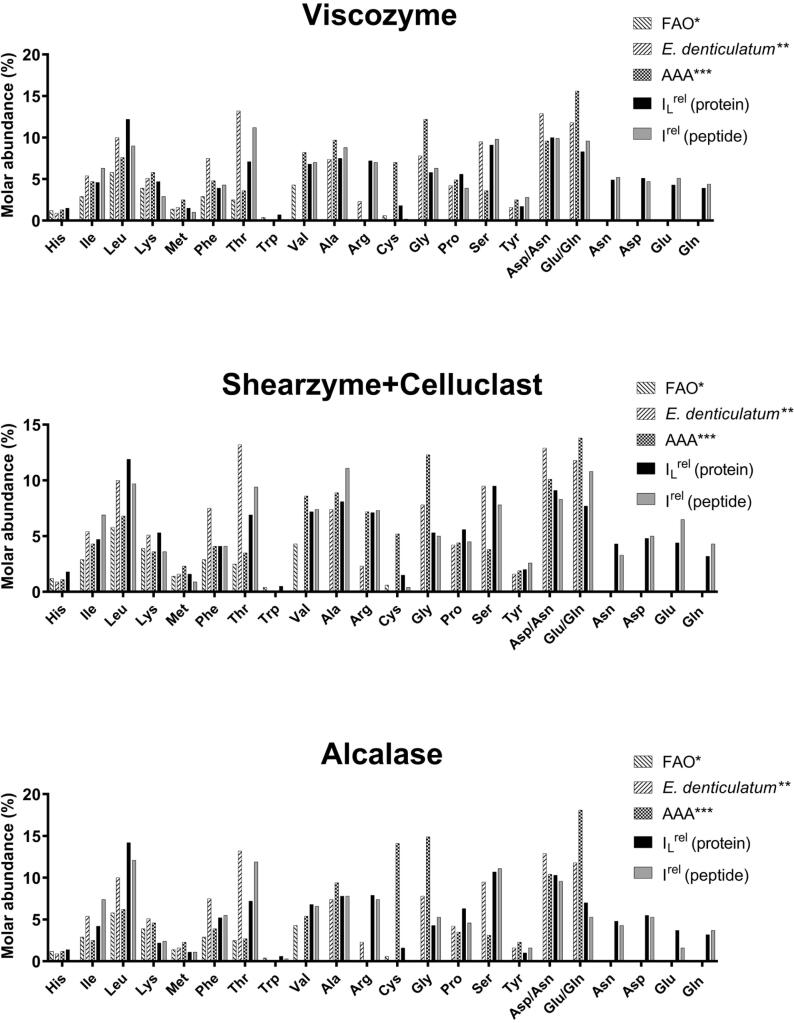
Amino acid (AA) composition (as relative molar abundance) for the three extracts using protein- and peptide-level based AA compositional analysis. For reference, minimum requirements according to the FAO* (WHO/FAO/UNU, 2007), AA analysis (AAA) of full *E. denticulatum* by LC-MS** (Naseri et al., 2019), and AAA of the extracts by LC-MS*** (Naseri et al., 2020).

In Fig. 3 (see Table S3 for numerical data), the AA compositions based on data for both semi-specific length-normalized protein-level analysis, I_L_^rel^ (s), and MS1 intensity-weighted peptide-level estimation, are presented for the three extracts. For comparison, the LC-MS-based AA composition for the extracts (Naseri et al., 2020), LC-MS based AA composition for the whole *E. denticulatum* (Naseri et al., 2019), and the minimum adult dietary requirements according to the WHO/FAO/UNO (WHO/FAO/UNU, 2007), are presented as well. Overall, similarity in the level of individual AAs was seen, but notable discrepancies were evident. Based on protein-level estimation, several AAs stand out as markedly different to the results obtained from extract AAA. The relative contents of Leu, Thr, Ser, Trp, and, for extracts V and S + C, Arg were notably higher, while the levels of Met, Cys, Gly, Asp/Asn, and Glu/Gln were markedly lower. The higher levels of Leu, Thr, Ser, and Trp were well in line with the limitations for conventional AAA presented above. That being said, protein-level abundance-based estimation is based on the full-length protein sequences. As the native proteins may differ significantly from the full length (e.g. pre- or pro-form) of the protein, this may influence the distribution. Furthermore, protein-level estimation also assumes that intact proteins are present in the extract, even when not fully supported by experimental data. In some cases, experimental sequence coverage may be very low, which could be the result of partial hydrolysis of the protein during the extraction process. In the particular case of Cys, the levels determined by AAA of the extracts was remarkably higher than those found in the AAA of the full organism (Naseri et al., 2019), indicating that this discrepancy may be a result of overestimation in the extract AAA.

Using peptide-level MS1 intensities to estimate AA composition, also showed deviations from results obtained by conventional AAA. Levels of Ile, Leu, Thr, and Ser were considerably higher which again may be a direct consequence of decreased quantitative recovery in AAA. Similarly to protein-level estimation, levels of Met, Cys, Gly, Asp/Asn, and Gln/Glu were considerably lower using peptide-level estimation. Additionally, levels of His and Trp were markedly lower by peptide-level estimation, while Met and Cys levels were lower compared to protein-level estimation. This observation is linked to the phenomenon of peptide detectability in bottom-up proteomics analysis. The probability of detecting a peptide by electrospray MS/MS as well as the intensity output of detected peptides relies highly on the AA sequence of the individual peptide (Jarnuczak et al., 2016, Serrano et al., 2019). Much in agreement with observations in this study, detectability of peptides with Met, His, Trp, and Cys has been reported to be reduced (Zhang & Jian, 2014). In a recent study (Jafarpour et al., 2020), a similar approach of peptide-level estimation was applied for characterization of protein hydrolysates from Atlantic cod. Here, a considerably better agreement between AAA and proteomics data was observed. This indicates that the somewhat poor quality of the protein extracts *E. denticulatum*, and therefore also low number of both peptide and protein identifications, may affect the applicability of using proteomics data for AA composition determination.

Regardless of applying protein- or peptide-level data for estimating the AA composition, the possibility of acquiring a full AA profile is evident. Nevertheless, systematic investigations of accuracy and robustness of the methodology are required. Moreover, further development in absolute, relative protein quantification as well as a better understanding of the peptide level sequence-intensity relationship, is required to unleash the full potential of such an approach. Taking into account the limitations of the different analytical approaches to determine AA composition, all enzymatic extracts appeared to comply very well to the WHO/FAO/UNU recommendations, highlighting their potential as dietary protein. However, as significant levels of potentially anti-nutritional factors such as lectins were detected, further processing of the protein extracts may be required for food applications. One such approach may be enzymatic hydrolysis, which may also result in the release of potentially functional and bioactive peptides as an added value to the nutritional properties.

### Identification and prediction of bioactive and functional peptides

Several abundant proteins (>2% I_L_^rel^) identified in this study contain peptides with verified *in vitro* emulsifying and antioxidant properties, as presented in Table 3. As seen, proteins embedding peptides of significantly high potential (indicated by *), were identified in very high abundances. Moreover, abundant proteins identified in this study also include several previously uncharacterized proteins in terms of embedding of potentially bioactive peptides. Using bioinformatic prediction of functional properties, the identified proteins dis indeed contain a multitude of potentially bioactive peptides as indicated by their high prediction scores. Based on the 15 abundant and previously uncharacterized proteins identified, >110,000 potentially bioactive peptides were predicted (Table S4). Hereof, >10,000 were predicted as emulsifiers, while >67,000 and approximately 39,000 were predicted as potential free radical scavengers (SCA) and metal chelators (CHE), respectively. This massive number was achieved even after applying scoring thresholds (Jafarpour et al., 2020) for emulsifier (z-normalized score > 2, indicating that the peptide scores higher than 97.5% of random peptides of the same length) and antioxidant peptides (SCA > 0.43 and CHE > 0.3, indicating that a peptide is more likely to be antioxidant than not). It should be noted that in these predictions, significant redundancy can be observed through sequence overlaps. For antioxidant peptides, clustering by sequence identity reduced the number of probable SCA and CHE peptides to 97 and 40 clusters, respectively (Table S4). Clustering of emulsifier peptides was not performed, although a high degree of sequence overlap can be observed (see Supplementary Data), which would also dramatically reduce the number of sequences using cluster representation. For instance, in the case of predicted β-emulsifiers, the fifth representative peptide (pEb-5) was only the 86th highest scoring peptide of all predicted β-emulsifiers. This means that the 85 higher scoring peptides represent merely four clusters, indicating the high level of sequence overlap in predictions. Nevertheless, the high level of clustering may also be an advantage in targeted hydrolysis for release of the peptides, as this can reduce the requirements for proteolytic specificity for obtaining functional peptides. If one peptide in the cluster, with a significantly high score, can be released, it has high likelihood of being functional. Although this would require *in vitro* validation and comparison of e.g. the highest scoring peptide in a cluster with the peptide(s) releasable through targeted hydrolysis, previous studies have shown that slight changes in peptide length and/or sequence does not necessarily impair function (García-Moreno et al., 2020, Yesiltas et al., 2021).

**Table 3:** Overview of previously verified and newly predicted emulsifier and antioxidant peptides from identified and abundant proteins in the three *E. denticulatum* extracts. Peptides are grouped by their predicted mode of function and listed with their annotated name, predicted score, parent protein(s) annotation, amino acid sequence, length (L), charge at pH 7 (*z*) as well as the abundance by length-normalized, relative intensity (I_L_^rel^) by semi-specific analysis for the three enzymatic extracts (Viscozyme® (V), Alcalase® (A), and Shearzyme®+Celluclast® (S + C), respectively).

Type^3^	Annotation	Score^4^	Protein(s)^5^	Sequence	L	*z*^6^	I_L_^rel^ (V)	I_L_^rel^ (A)	I_L_^rel^ (S + C)
Verified emulsifier peptides^1^									
α	80-S-A*	3.3	c7052_g1_i1, c7052_g1_i2	IGYTVRNSLRVTVRDLSNLGLILDALVR	28	2	12.9%	24.5%	9.6%
	81-S-A	2.9	c7052_g1_i1	AVKDAVRRATLLTKAAGTGLGKVLS	25	4	8.6%	14.6%	6.9%
β	83-S-B*	3.18	c7052_g1_i1, c7052_g1_i2	RAGSNSLSRISFGISNEADLRDQAR	25	1	12.9%	24.5%	9.6%
	84-S-B*	2.75	c6313_g1_i1	VGFACSGSAQTYLSFEGDNTGRGEEEVAI	29	−4.1	1.3%	5.7%	1.1%
	85-S-B	3.27	c7052_g1_i1	LSIREGGRSTGGFSAQVRAR	20	3	8.6%	14.6%	6.9%
	86-S-B*	5.43	c7052_g1_i1	ELQVSARVTLEIEL	14	−2	8.6%	14.6%	6.9%
γ	87-S-G	5.72	c1505_g2_i1	RELQRDDNVRNVRILLSSLVLLLDWLVCLL	30	−0.1	27.4%	30.8%	26.4%
	88-S-G	5.06	c1505_g2_i1	AVLVVCLQQVRELQRDDNVRN	21	−0.1	27.4%	30.8%	26.4%

Predicted emulsifier peptides									
α	pEa-1	3.77	c1545_g1_i1	VKRISGLIYEETRNVLKVF	19	2	2.8%	0.0%	7.4%
	pEa-2	3.64	c13559_g1_i1 c6825_g1_i1	IYKVLKQV	8	2	2.2%	0.0%	8.1%
	pEa-3	3.62	c10861_g1_i1	RFFLRVVRGVRQKV	14	5	0.5%	3.7%	0.6%
	pEa-4	3.44	c14987_g1_i1**	VQKLSRVID	9	1	0.0%	0.0%	0.0%
	pEa-5	3.37	c6825_g1_i1	VADLFERIASEAAKL	15	−1	2.2%	0.0%	8.1%
β	pEb-1	4.80	c7216_g1_i1	VRIRVDCK	8	1.9	2.5%	5.4%	2.8%
	pEb-2	4.12	c14987_g1_i1**	RLNCKID	7	0.9	0.0%	0.0%	0.0%
	pEb-3	3.72	c4419_g1_i1	LKVELNSGGQMR	12	1	1.7%	0.0%	2.0%
	pEb-4	3.55	c907_g1_i1	NIQKESTLHLVLRLRGGL	18	2.1	15.1%	5.3%	6.6%
	pEb-5	3.20	c13533_g1_i1	AKVRVTC	7	1.9	2.2%	0.0%	4.3%
γ	pEg-1	5.20	c13533_g1_i1	ITTVLALVCVITQVMQASANEQEHVHEHEH	30	−3.7	2.2%	0.0%	4.3%
	pEg-2	5.11	c24_g1_i1.p2	ILLVLCLSWLRRKVCRNHR	19	5	0.0%	3.4%	0.2%
	pEg-3	5.10	c10861_g1_i1	EREYELQKEFATLVLAVV	18	−2	0.5%	3.7%	0.6%
	pEg-4	4.69	c17933_g1_i1	FLLVFFFFFTDEDT	14	−3	5.2%	13.5%	6.2%
	pEg-5	4.43	c907_g1_i1	QDQQRLIFA	9	0	15.1%	5.3%	6.6%

Verified antioxidant peptides^2^									
SCA	120-S-SCA	0.49	c6313_g1_i1	RYVWN	5	1	1.3%	5.7%	1.1%
	123-S-SCA*	0.38	c6313_g1_i1	DFPVR	5	0	1.3%	5.7%	1.1%
	124-S-SCA*	0.43	c17304_g1_i1	AGDWLIGDR	9	−1	9.8%	3.9%	2.3%

Predicted antioxidant peptides									
SCA	pSCA-1	0.72	c6963_g1_i1	DFYAYIVFTWAGYHGVDLAKNKIASDF	27	−0.9	2.7%	0.9%	5.2%
	pSCA-2	0.71	c24_g1_i1	WYHY	4	0.1	0.0%	3.4%	0.2%
	pSCA-3	0.66	c10861_g1_i1	LQGSKFAVVEYGGIVDPILGLQP	23	−1	0.5%	3.7%	0.6%
	pSCA-4	0.64	c13533_g1_i1	ASANEQEHVHEHEHIIRTFS	20	−2.6	2.2%	0.0%	4.3%
	pSCA-5	0.64	c4419_g1_i1	VVPFSSWYAEQQRI	14	0	1.7%	0.0%	2.0%
CHE	pCHE-1	0.39	c17933_g1_i1	FFFFTDEDTFPSGPSLTTFCSP	22	−3.1	5.2%	13.5%	6.2%
	pCHE-2	0.36	c6405_g1_i2**	FAISLFRIFPASFMFMPFTH	20	1.1	0.0%	0.0%	0.0%
	pCHE-3	0.35	c6405_g1_i2**	PHPN	4	0.1	0.0%	0.0%	0.0%
	pCHE-4	0.34	c7216_g1_i1	FFSALLLLMNFPSPTMSLCTDDD	23	−3.1	2.5%	5.4%	2.8%
	pCHE-5	0.34	Multiple***	HP	2	0.1	4.2%	0.1%	9.9%

*Peptides were in their respective studies highlighted as particularly promising based on *in vitro* functional validation.

**c14987_g1_i1 and c6405_g1_i2 were only identified by a single peptide each and only through unspecific analysis (but in all samples) and the peptides are regarded as potential contaminants and thus protein identification as doubtful.

***The dipeptide can be found in five abundant proteins (c13559_g1_i1; c24_g1_i1; c6405_g1_i2; c6656_g1_i1; and c6825_g1_i1).

1 Verified *in vitro* emulsifying activity (Yesiltas et al., 2021).

2 Verified *in vitro* antioxidant activity (Submitted manuscript, Food Chemistry).

3 Type refers to mode of predicted function. For emulsifier peptides, α refers to peptides with predicted amphiphilic helical conformation, β refers to predicted amphiphilic sheet conformation, and γ refers to peptides with one end being mostly hydrophobic and the other mostly hydrophilic. For antioxidant peptides, SCA refers to predicted free radical scavengers while CHE refers to predicted metal chelators.

4 Predicted scores for emulsifier and antioxidant peptides were computed using EmulsiPred (García-Moreno et al., 2020, García-Moreno et al., 2020) and AnOxPePred (Olsen et al., 2020), respectively.

5 Protein annotation in accordance with the *de novo* protein database previously reported (Gregersen et al., 2020).

6 Net charge at pH = 7 was calculated using the peptide property calculator (Innovagen AB, Lund, Sweden).

As seen from Table 3, all predicted emulsifier peptides, using top-5 non-redundant representation, have z-normalized scores > 3, which means they score higher than 99.9% of random peptides, using the algorithm. This further indicated that, based only on computed amphiphilicity, the peptides are very likely to have emulsifying properties *in vitro*. However, two of the predicted and high scoring α-peptides (pEa-2 and pEa-4) were shorter than 10 AAs. It was previously suggested that for efficient interfacial stabilization in emulsions, peptides in an amphiphilic helix conformation should preferably be 15 AAs or longer (García-Moreno et al., 2020). This suggests that the two peptides, are not likely to be good α-emulsifiers, and further highlights a potential point for improvement of the predictive model. The optimal length range for peptides expected to form β-sheets at the interface, has been suggested to be 9–16 AAs (García-Moreno et al., 2020, Yesiltas et al., 2021). Four of the five predicted β-peptides, pEb-3 being the exception, fall just outside this range. Although such strict length requirements may not be the only governing parameter, additional (high scoring) peptides adhering to the size range can be found within their respective clusters. Moreover, all extracts contain high abundance proteins, which were previously shown to embed highly potent emulsifier peptides. In particular, four of the eight previously characterized peptides (80-S-A, 83-S-B, 84-S-B, and 86-S-B) displayed remarkable emulsifying properties (Yesiltas et al., 2021). This finding further adds to the potential of enzymatic *E. denticulatum* protein extracts as a high potential source of functional ingredients through e.g. targeted enzymatic hydrolysis.

The newly identified proteins suggest a high potential for release of antioxidant peptides. In fact, predicted peptides score higher than any previously reported (and validated) peptides, predicted by AnOxPePred; both in terms of SCA- and CHE-peptides (Jafarpour et al., 2020, Olsen et al., 2020, Thaha et al., 2021), (Submitted manuscript, Food Chemistry). In addition, multiple previously *in vitro* validated radical scavenger peptides were identified. Of these, particularly two peptides (123-S-SCA and 124-S-SCA) showed exceptional *in vitro* potential for retarding lipid oxidation in emulsions stabilized by Tween20 (Submitted manuscript, Food Chemistry). As the predicted scores of all peptides representing the top5 clusters were markedly higher, this indicates that these *E. denticulatum* extracts may indeed be exceptional sources of antioxidant peptides. Furthermore, two of the predicted antioxidant peptides (pSCA-4 and pCHE-1) showed significant sequence overlap with predicted emulsifier peptides (pEg-1 and pEg-4, respectively), indicating that hydrolysis could potentially result in multifunctional peptides, thereby further adding to the potential of the protein extracts. Regardless, validation *in vitro* would be a logical next step to verify their potential as functional and bioactive peptides.

## Conclusion

As the hunt for new and sustainable protein sources intensifies, the requirements for sustainable extraction methods follow. In contrast to evaluating an extraction method simply by means of estimating protein yield and content through classical methods such as Dumas and Kjeldal nitrogen, more in-depth and protein-specific analysis can bring significant added value as a base for informed decision making. In this study, we have, for three enzymatic protein extracts from *E. denticulatum*, demonstrated how Dumas-N may significantly overestimate protein content compared to conventional AAA by LC-MS and how a simple protein assay (Qubit) may be used as a more accurate method. Moreover, we present a workflow where combining quantitative proteomics with bioinformatic analysis can be used as a very powerful method for evaluating methods for protein extraction and cellular lysis. The predicted subcellular localization was used to evaluate extraction efficiency, and the combination of Shearzyme® and Celluclast® extracted more protein, due to more efficient cell lysis compared to singular added Alcalase® and Viscozyme®. That combination of Shearzyme® and Celluclast® increased intracellular protein recovery as well as total protein content in the obtained extracts.

Furthermore, the applied methodology identified and predicted both known and novel functional or bioactive peptides embedded in high abundance proteins. >110,000 potential emulsifier and antioxidant peptides were predicted across newly identified and abundant proteins. By clustering of peptides based on sequence overlap, these peptides represent regions of the abundant proteins, which have high potential for application as functional food ingredients and may be released through targeted enzymatic hydrolysis. Finally, we demonstrated that, bottom-up proteomics analysis may in the future not just compliment but potentially replace conventional AAA. Ultimately, proteomics analysis has the potential to, using a standardized workflow and one-shot analysis, replace a multitude of conventional analytical methods applied in the food protein field, while simultaneously adding several additional dimensions to the extractable information.

## Funding

This work was, in part, supported by the Innovation Fund Denmark (Grant number 7045-00021B (PROVIDE)).

## Declaration of Competing Interest

The authors declare that they have no known competing financial interests or personal relationships that could have appeared to influence the work reported in this paper.
