# Supplementary material for: Enzymatic extraction improves intracellular protein recovery from the industrial carrageenan seaweed Eucheuma denticulatum revealed by quantitative, subcellular protein profiling: A high potential source of functional food ingredients
Source: Food Chem X. 2021 Oct 20;12:100137. doi: 10.1016/j.fochx.2021.100137 (PMC8554166; doi:10.1016/j.fochx.2021.100137)
Supplement: Supplementary data 1 [file mmc1.docx]

**Supplementary Information for**

**Enzymatic extraction improves intracellular protein recovery from the industrial carrageenan seaweed *Eucheuma denticulatum* revealed by quantitative, subcellular protein profiling: A high potential source of functional food ingredients**

Simon GREGERSEN^a*^, Anne-Sofie Havgaard KONGSTED^a^, Rikke Brønnum NIELSEN^a^, Søren Storck HANSEN^a^, Frederik Andersen LAU^a^, Jacob Bisgaard RASMUSSEN^a^, Susan Løvstad HOLDT^b^, Charlotte JACOBSEN^b^

^a^ Department of Chemistry and Bioscience, Aalborg University, Denmark

^b^ National Food Institute, Technical University of Denmark, Denmark

*Correspondence: [sgr@bio.aau.dk](mailto:sgr@bio.aau.dk)

Quality-based filtering of identified proteins

Following the initial and conventional filtering of contaminants and reverse (false positives) from the identified protein (groups), an additional round of quality-based filtering was applied. The methodology largely adheres to the principles previously described (Gregersen et al., 2020), and was conducted as follows. A protein group is filtered before final re-quantification if it falls below the threshold in at least 3 of 5 of the criteria specified below:

1. A protein group is only identified by one unique peptide across all 18 combinations of samples and analytical conditions (max_uni =< 1)
2. The maximum sequence coverage of a protein group across all 18 combinations of samples and analytical conditions is lower than 5% (Max_SC =< 5)
3. The maximum score of a protein group across all 18 combinations of samples and analytical conditions is lower than 40 (Score_max < 40)
4. The total amount of acquired MS/MS spectra for a protein group across all 18 combinations of samples and analytical conditions is lower than 10 (MS/MS_sum < 10)
5. A protein group is only quantified in one sample replicate under one applied analytical condition (Count_IDs =< 1)


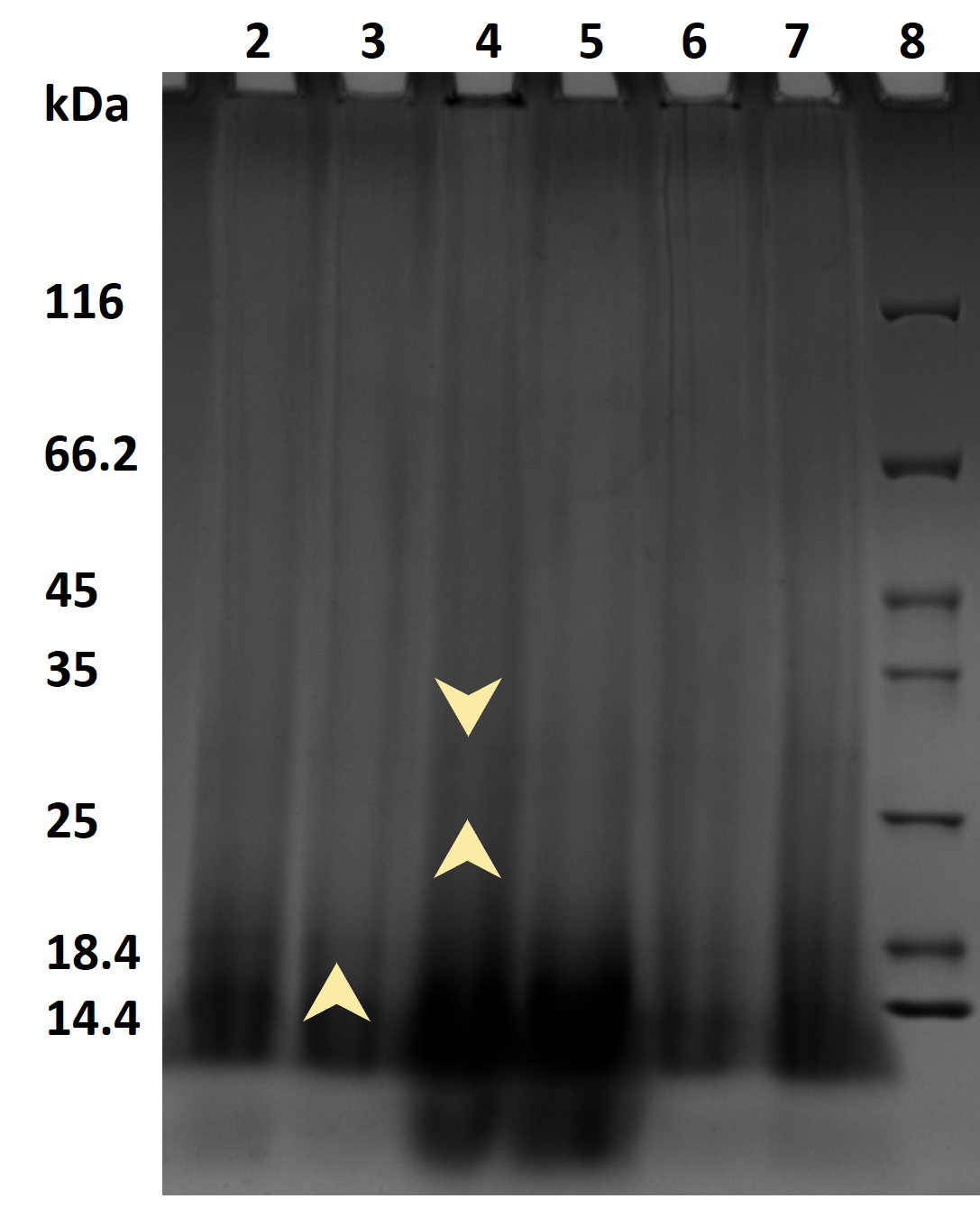


Figure S1: SDS-PAGE page analysis (4-20% gradient Bis-Tris) of the extracts solubilized in the SDS-ABC buffer. Lanes 2-7 show the extracts (V1, V2, A1, A2, S+C1, and S+C2, respectively) while lane 8 is the MW marker. Arrows indicate location of the vague bands.

Table S1: Summary of protein group identifications for initial proteomics analysis (top) and after quality-based data filtering (bottom), showing the number of identified protein groups for each replicate and the total number unique protein groups across both replicates for each extraction method (total) for the individual analysis methods applied. The number of shared identifications between replicates (replicate ID) and the relative share of duplicates (duplicate ID / Total) and one replicate (100% - duplicate share) identifications are shown for each extraction method.

|  | Viscozyme | | | Alcalase | | | Shearzyme+Celluclast | | |
| --- | --- | --- | --- | --- | --- | --- | --- | --- | --- |
|  | V-1 | V-2 | Total | A-1 | A-2 | Total | S+C-1 | S+C-2 | Total |
| Tryptic | 78 | 54 | 80 | 35 | 29 | 43 | 83 | 85 | 94 |
| Semi-specific | 67 | 51 | 72 | 33 | 24 | 38 | 70 | 78 | 84 |
| Unspecific | 62 | 45 | 65 | 34 | 25 | 35 | 65 | 69 | 79 |
|  | Duplicate ID | Duplicate share | One replicate share | Duplicate ID | Duplicate share | One replicate share | Duplicate ID | Duplicate share | One replicate share |
| Tryptic | 51 | 64% | 36% | 21 | 49% | 51% | 74 | 79% | 21% |
| Semi-specific | 46 | 64% | 36% | 19 | 50% | 50% | 64 | 76% | 24% |
| Unspecific | 42 | 65% | 35% | 24 | 69% | 31% | 55 | 70% | 30% |
| After quality-based filtering | | | | | | | | | |
|  | V-1 | V-2 | Total | A-1 | A-2 | Total | S+C-1 | S+C-2 | Total |
| Tryptic | 61 | 47 | 64 | 29 | 22 | 32 | 63 | 66 | 67 |
| Semi-specific | 54 | 45 | 58 | 28 | 21 | 31 | 57 | 62 | 63 |
| Unspecific | 50 | 41 | 53 | 28 | 22 | 29 | 55 | 56 | 61 |
|  | Duplicate ID | Duplicate share | One replicate share | Duplicate ID | Duplicate share | One replicate share | Duplicate ID | Duplicate share | One replicate share |
| Tryptic | 44 | 69% | 31% | 19 | 59% | 41% | 62 | 93% | 7% |
| Semi-specific | 41 | 71% | 29% | 18 | 58% | 42% | 56 | 89% | 11% |
| Unspecific | 38 | 72% | 28% | 21 | 72% | 28% | 50 | 82% | 18% |


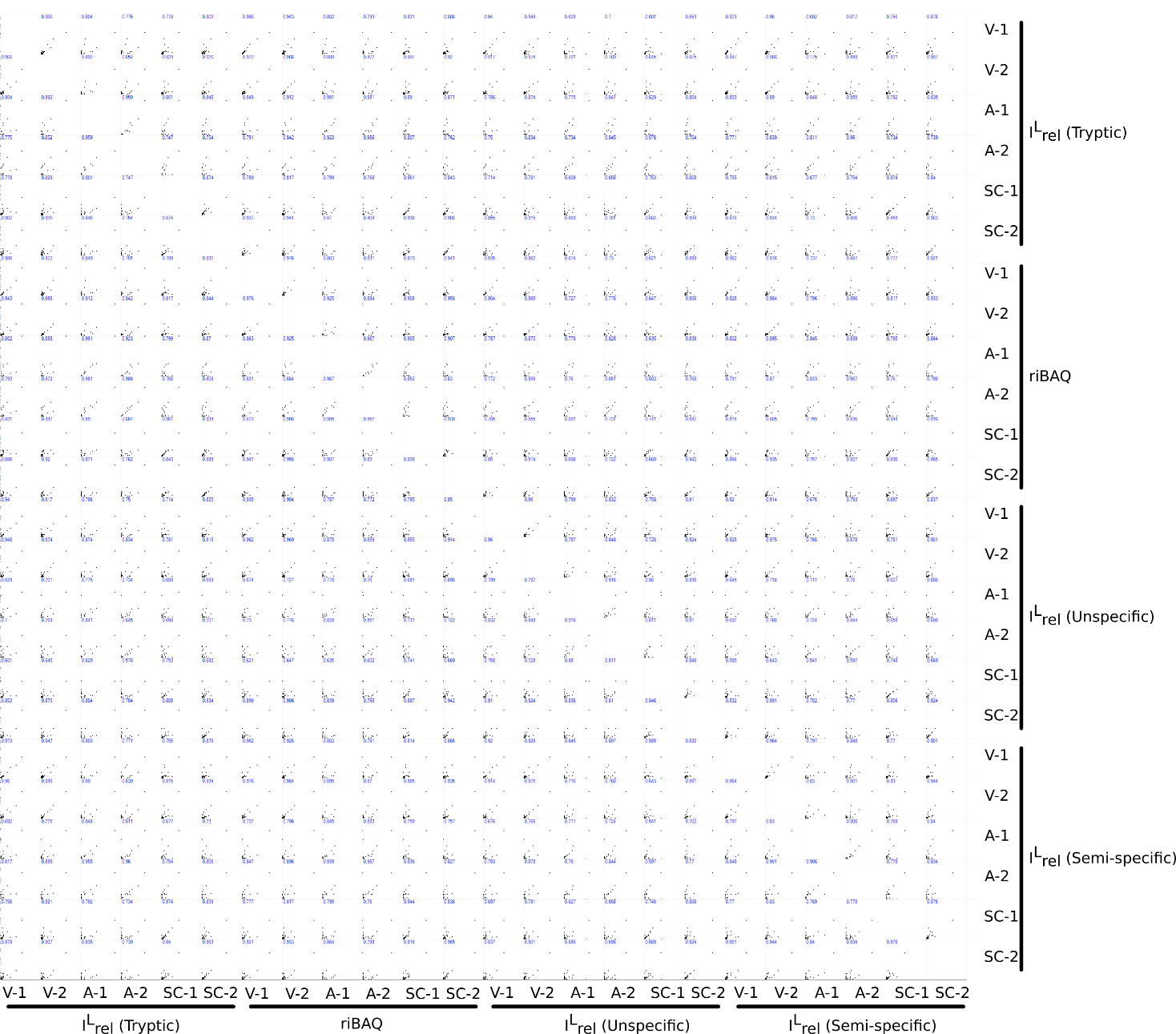


Figure S2: Scatter plots of initial, individual relative quantification for all individual extract replicates and all applied analysis methods. The figure is attached in high resolution in the supplementary data.


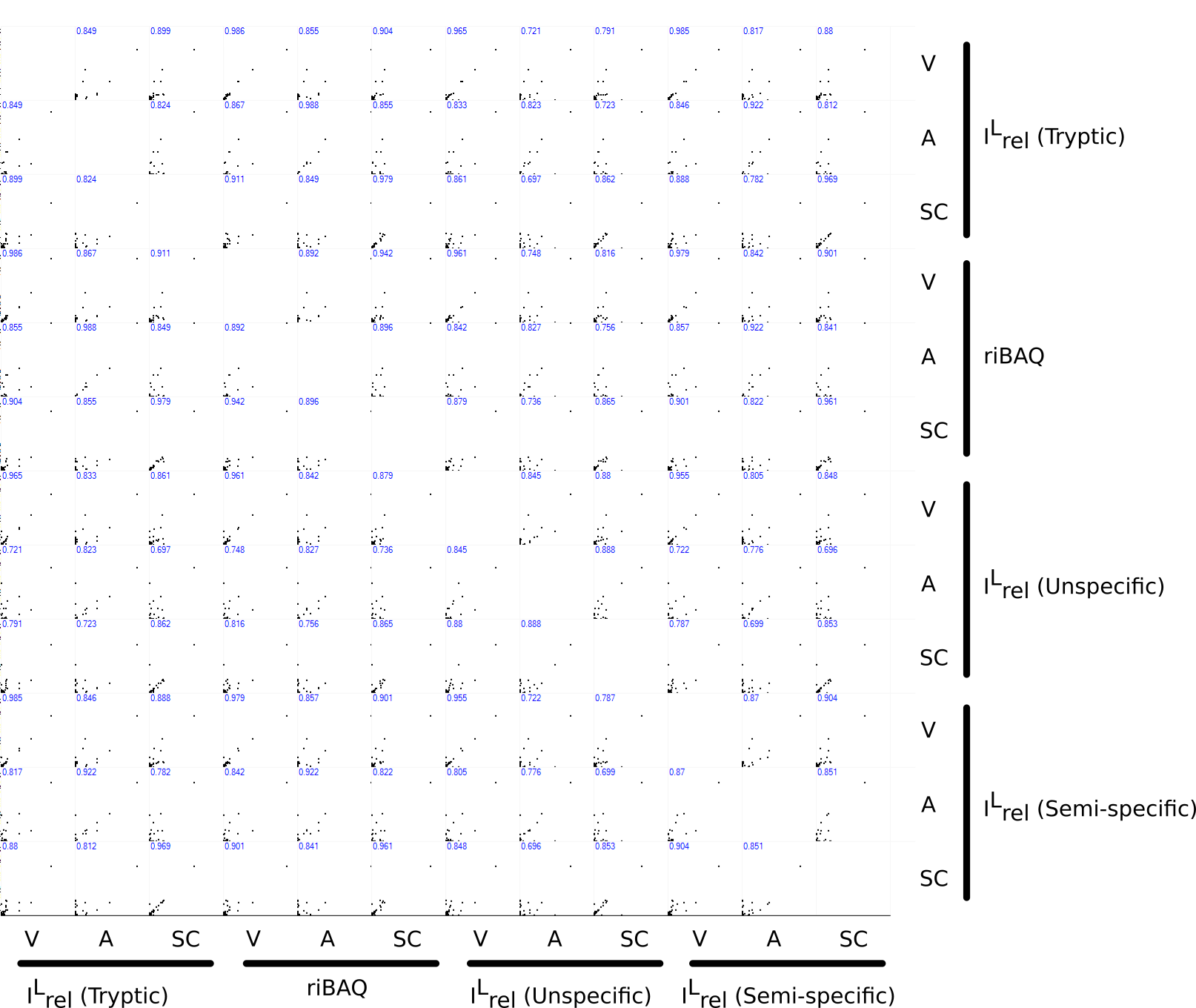


Figure S3: Scatter plots for initial quantification with requirement of duplicate identification and plotted as mean relative abundances for extract duplicates. The figure is attached in high resolution in the supplementary data.


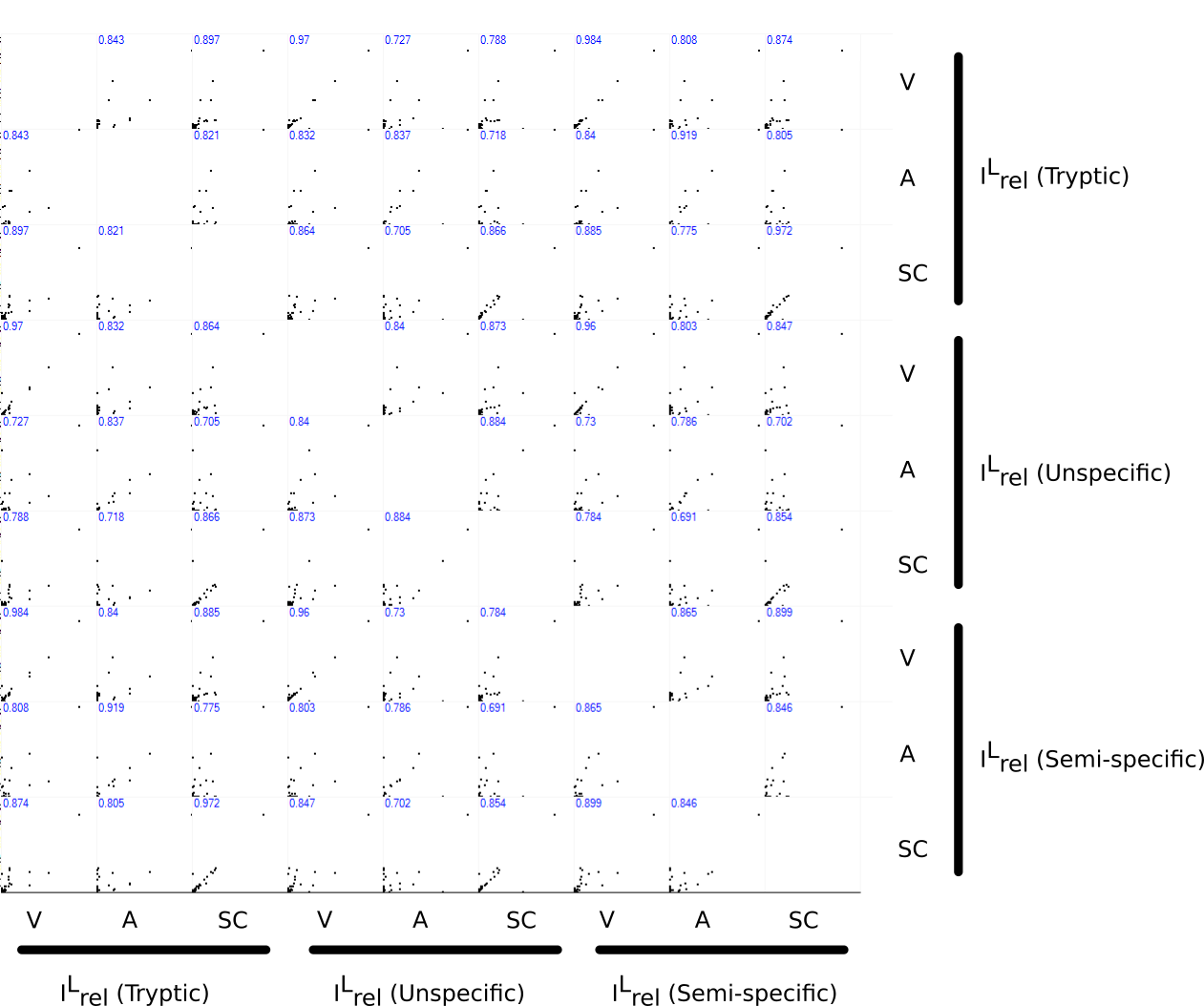


Figure S4: Scatter plots for re-quantified protein groups following quality-based filtering with requirement of duplicate identification and plotted as mean relative abundances for extract duplicates. The figure is attached in high resolution in the supplementary data.


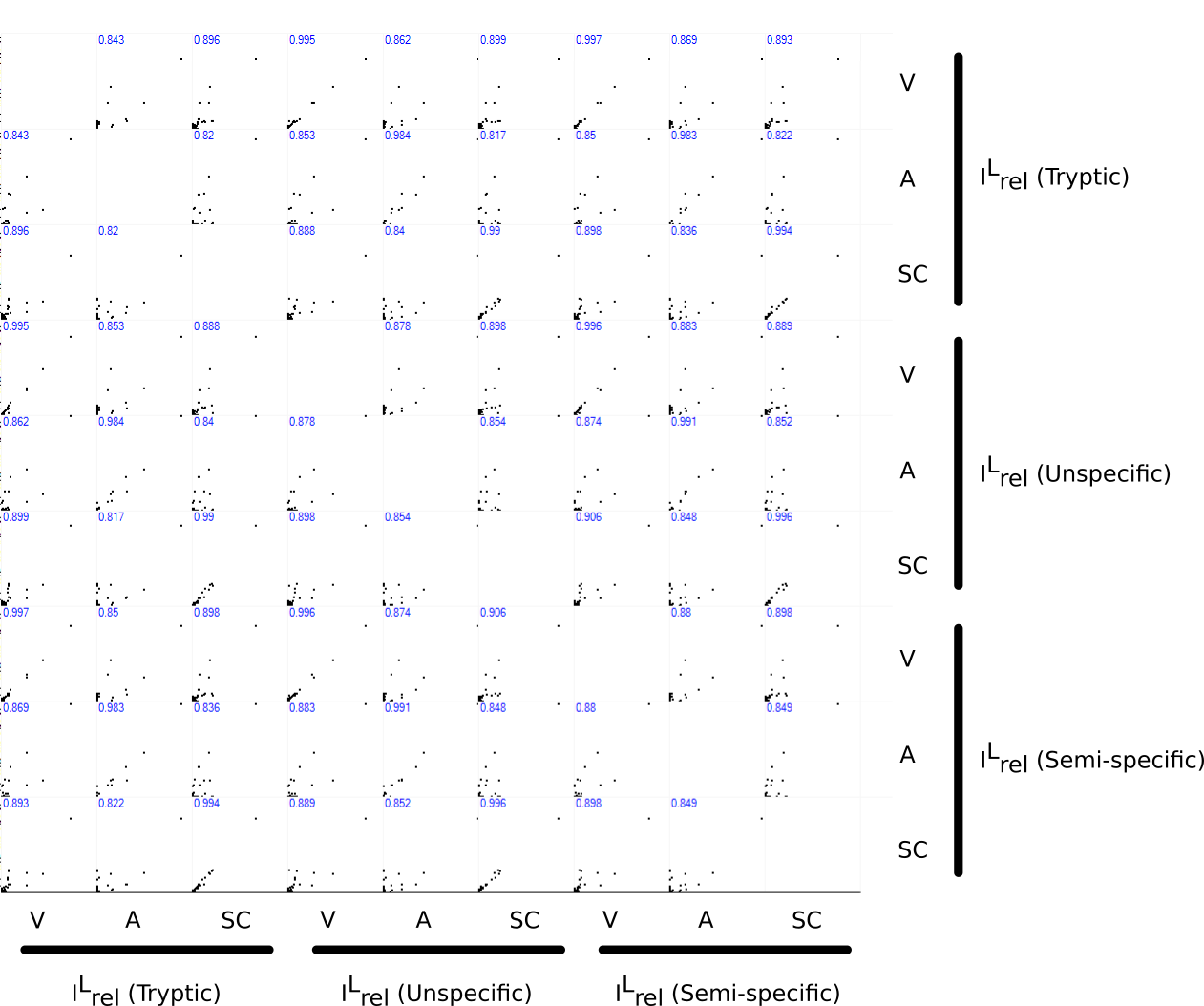


Figure S5: Scatter plots for re-quantified protein groups following quality-based filtering and removal of suspect outliers, with requirement of duplicate identification and plotted as mean relative abundances for extract duplicates. The figure is attached in high resolution in the supplementary data.

Table S2: Summary of BLAST analysis from UniProt for the selected, abundant proteins identified across extracts. Listed by the protein ID, the UniProt AC# of the BLAST target, the BLAST score, and % identity with and details regarding the BLAST target.

| Protein ID | BLAST target AC# | BLAST score | Identity and target information |
| --- | --- | --- | --- |
| c10861_g1_i1.p1 | Q95LI2 | 83 | 36% identity with Vitrin (Bos taurus) |
| c13533_g1_i1.p1 | Q7VH33 | 66 | 29.3% identity with 3-isopropylmalate dehydrogenase (Helicobacter hepaticus) |
| c13559_g1_i1.p1;  c6825_g1_i1.p3 | Q64475  Q75VN4 | 432  417 | 74.8% identity with Histone H2B type 1-B (Mus musculus)  69.8% identity with Histone H2B (Zhangixalus schlegelii) |
| c14987_g1_i1.p1 | Q93Y35 | 900 | 52.4% identity with 26S proteasome non-ATPase regulatory subunit 6 homolog (Arabidopsis thaliana) |
| c1505_g2_i1.p1 | C8V7C6 | 84 | 42.1% identity with Conserved oligomeric Golgi complex subunit 6 (Emericella nidulans) |
| c1545_g1_i1.p5 | Q8T7J8 | 499 | 96.1% identity with Histone H4 in Eimeria tenella |
| c17304_g1_i1.p1 | P84331 | 1393 | 95.1% identity with Lectin ESA-2 (Eucheuma serra) |
| c17933_g1_i1.p1 | Q8LPN7 | 76 | 31.2% identity with E3 ubiquitin-protein ligase RING1-like (Arabidopsis thaliana) |
| c24_g1_i1.p2 | Q66628 | 82 | 22.4% identity with Major capsid protein (Equine herpesvirus 2 (strain 86/87) (EHV-2) |
| c4419_g1_i1.p2 | P16569 | 197 | 59.4% identity with Phycobilisome 7.8 kDa linker polypeptide, allophycocyanin-associated, core (Microchaete diplosiphon) |
| c6313_g1_i1.p1 | n/a | n/a | No BLAST hits |
| c6405_g1_i2.p4 | Q8TGK9 | 183 | 67.3% identity with Putative uncharacterized protein YPL135C-A (Saccharomyces cerevisiae) |
| c6656_g1_i1.p1 | Q944G9 | 1086 | 55.7% identity with Fructose-bisphosphate aldolase 2, chloroplastic (Arabidopsis thaliana) |
| c6963_g1_i1.p1 | P15214 | 320 | 35.8% identity with Glutathione S-transferase GST-6.0 (Proteus mirabilis) |
| c7052_g1_i1.p1 | P0A3U9 | 303 | 34.8% identity with 26 kDa periplasmic immunogenic protein (Brucella suis biovar 1) |
| c7052_g1_i2.p1 | P0A3U9 | 331 | 36.7% identity with 26 kDa periplasmic immunogenic protein (Brucella suis biovar 1) |
| c7216_g1_i1.p1 | Q9W770 | 167 | 28.6% identitty with Spondin-1 (Gallus gallus) |
| c7502_g1_i1.p1 | Q6CK59 | 492 | 78.8% identity with Histone H2A (Kluyveromyces lactis (strain ATCC 8585 / CBS 2359 / DSM 70799 / NBRC 1267 / NRRL Y-1140 / WM37) (Yeast) (Candida sphaerica)) |
| c907_g1_i1.p2 | P0CH07 | 566 | 85.9% identity with Ubiquitin-60S ribosomal protein L40 (Ubiquitin-60S ribosomal protein L40) |


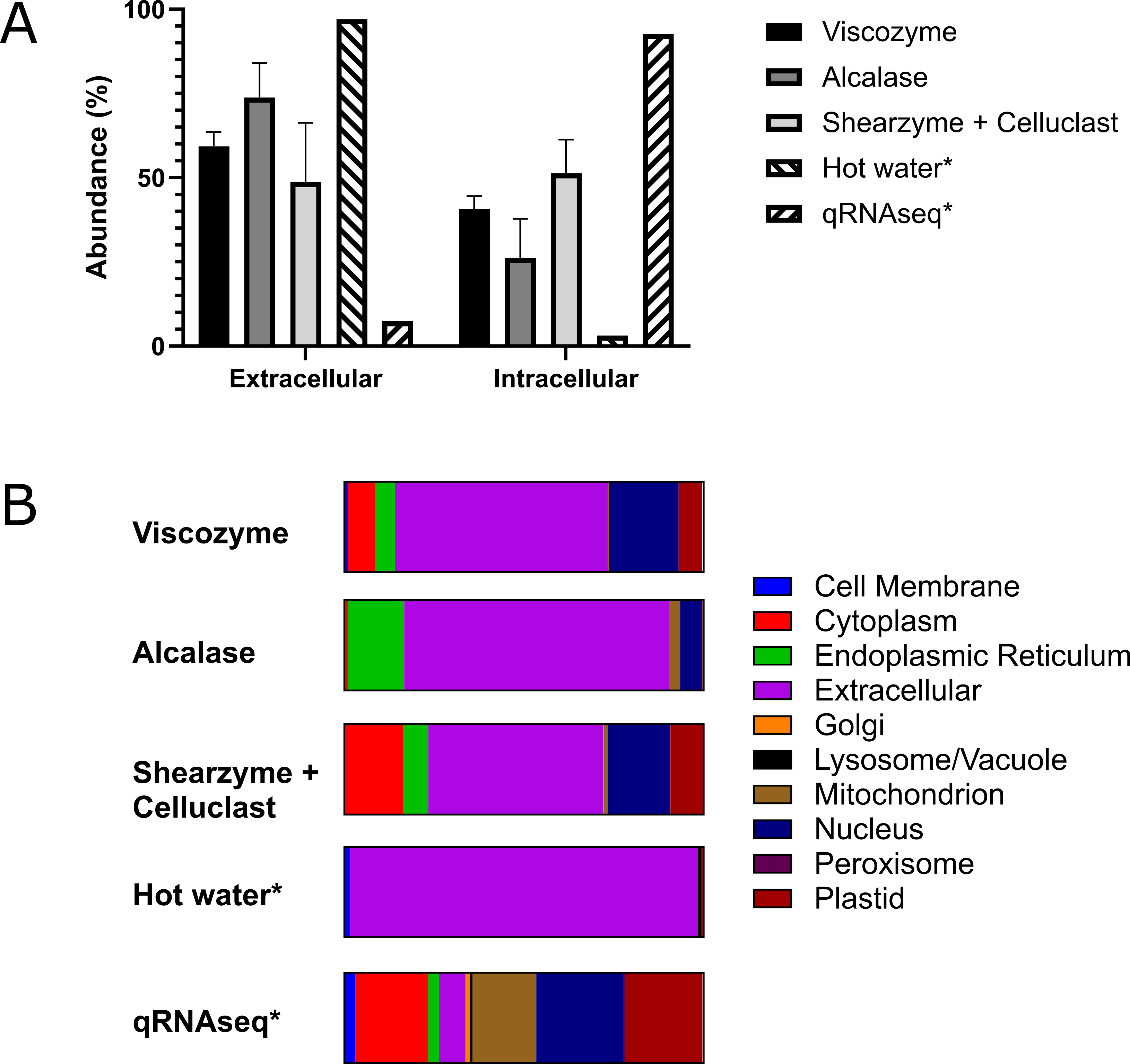


Figure S6: Distribution of quantified proteins according to predicted subcellular localization evaluated by semi-specific I_L_^rel^ before manual filtering of suspect outliers. A: Relative proportion of proteins predicted as extracellular and proteins allocated to the binned compartment “intracellular” (i.e. non-extracellular) by extraction method. B: Relative proportion of proteins allocated to each subcellular compartment as predicted by DeepLoc (Almagro Armenteros, Sønderby, Sønderby, Nielsen, & Winther, 2017), presented as a stacked bar plot for each extraction method. *According to (Gregersen et al., 2020)

Figure S7: Relative subcellular distribution (by I_L_^rel^) of identified proteins by analysis method after (A) and before (B) manual filtering of suspect outliers.

Table S3: Amino acid (AA) composition (as relative molar abundance) for the three extracts using protein- and peptide-level based AA compositional analysis. For reference, minimum requirements according to the FAO^1^ (WHO/FAO/UNU, 2007), AA analysis (AAA) of full *E. denticulatum* by LC-MS^2^ (Naseri, Holdt, & Jacobsen, 2019), and AAA of the extracts by LC-MS^3^ (Naseri et al., 2020) are shown.

|  | |  |  | Viscozyme | | | Alcalase | | | Shearzyme+Celluclast | | |
| --- | --- | --- | --- | --- | --- | --- | --- | --- | --- | --- | --- | --- |
| Amino acid | | FDA^1^ | AAA^2^ | AAA^3^ | Semispc  (pro) | Semispc  (pep) | AAA^3^ | Semispc  (pro) | Semispc  (pep) | AAA^3^ | Semispc  (pro) | Semispc  (pep) |
| His | | 1.2% | 0.9% | 1.3% | 1.5% | nd. | 1.2% | 1.4% | nd. | 1.1% | 1.8% | nd. |
| Ile | | 2.9% | 5.4% | 4.7% | 4.6% | 6.3% | 2.5% | 4.2% | 7.4% | 4.3% | 4.7% | 6.9% |
| Leu | | 5.8% | 10.0% | 7.6% | 12.2% | 9.0% | 6.2% | 14.2% | 12.1% | 6.8% | 11.9% | 9.7% |
| Lys | | 3.9% | 5.1% | 5.8% | 4.7% | 2.9% | 4.6% | 2.2% | 2.4% | 3.6% | 5.3% | 3.6% |
| Met | | 1.4% | 1.6% | 2.5% | 1.5% | 1.0% | 2.3% | 1.1% | 1.1% | 2.3% | 1.6% | 0.9% |
| Phe | | 2.9% | 7.5% | 4.8% | 3.9% | 4.3% | 3.9% | 5.2% | 5.5% | 4.1% | 4.1% | 4.1% |
| Thr | | 2.5% | 13.2% | 3.6% | 7.1% | 11.2% | 2.7% | 7.2% | 11.9% | 3.5% | 6.9% | 9.4% |
| Trp^4^ | | 0.4% | n/a | n/a | 0.7% | 0.1% | n/a | 0.6% | 0.3% | n/a | 0.5% | nd. |
| Val | | 4.3% | 0.0% | 8.2% | 6.8% | 7.0% | 5.4% | 6.8% | 6.6% | 8.6% | 7.2% | 7.4% |
| Ala | |  | 7.4% | 9.7% | 7.5% | 8.8% | 9.4% | 7.8% | 7.8% | 8.9% | 8.1% | 11.1% |
| Arg | |  | 2.3% | nd. | 7.2% | 7.0% | nd. | 7.9% | 7.4% | 7.2% | 7.1% | 7.3% |
| Cys^5^ | |  | 0.1% | 7.0% | 1.8% | 0.2% | 14.1% | 1.6% | 0.1% | 5.2% | 1.5% | 0.4% |
| Gly | |  | 7.8% | 12.2% | 5.8% | 6.3% | 14.9% | 4.3% | 5.3% | 12.3% | 5.3% | 5.0% |
| Pro | |  | 4.2% | 4.9% | 5.6% | 3.9% | 3.5% | 6.3% | 4.6% | 4.4% | 5.6% | 4.5% |
| Ser | |  | 9.5% | 3.6% | 9.1% | 9.8% | 3.1% | 10.7% | 11.1% | 3.8% | 9.5% | 7.8% |
| Tyr | |  | 1.6% | 2.5% | 1.7% | 2.8% | 2.3% | 1.0% | 1.6% | 1.9% | 2.0% | 2.6% |
| Asp/Asn^6^ | |  | 12.9% | 9.6% | 10.0% | 9.9% | 10.4% | 10.3% | 9.6% | 10.1% | 9.1% | 8.3% |
| Glu/Gln^6^ |  | | 11.8% | 15.6% | 8.3% | 9.6% | 18.1% | 7.0% | 5.3% | 13.8% | 7.7% | 10.8% |
| Asn^6^ | |  | n/a | n/a | 4.9% | 5.2% | n/a | 4.8% | 4.3% | n/a | 4.3% | 3.3% |
| Asp^6^ | |  | n/a | n/a | 5.1% | 4.7% | n/a | 5.5% | 5.3% | n/a | 4.8% | 5.0% |
| Glu^6^ | |  | n/a | n/a | 4.3% | 5.1% | n/a | 3.7% | 1.6% | n/a | 4.4% | 6.5% |
| Gln^6^ | |  | n/a | n/a | 3.9% | 4.4% | n/a | 3.2% | 3.7% | n/a | 3.2% | 4.3% |
| EAA/NEAA^7^ | | | 0.76 | 0.59 | 0.57 | 0.54 | 0.38 | 0.58 | 0.70 | 0.51 | 0.61 | 0.55 |

Essential AAs are highlighted by light grey shaded background. “nd”: not detected under the analytical conditions. “n/a”: Obtaining this data point is not possible under the given condition in the applied analytical method. ^4^Trp is fully degraded during the acid hydrolysis applied in AAA. ^5^Cys is detected in a dimer form (cysteine) and disulfide bonds remain intact during acid hydrolysis in AAA. Cysteine content has been converted to reflect monomeric cysteine content. ^6^In AAA, it is not possible to distinguish Asp from Asn and Glu from Gln, due to deamidation of Asn and Gln. Consequently, using AAA it is only possible to report as a sum of both species, while LC-MS/MS makes it possible to distinguish.

Table S4: Summary statistics for all predicted emulsifier and antioxidant peptides from abundant proteins identified in this study. Peptides were predicted using EmulsiPred (García-Moreno, Gregersen, et al., 2020; García-Moreno, Jacobsen, et al., 2020) and AnOxPePred (Olsen et al., 2020), respectively.

|  | Emulsifier peptides | | | | Antioxidant peptides | | | |
| --- | --- | --- | --- | --- | --- | --- | --- | --- |
|  | α | β | γ | Sum | SCA | | CHE | |
| Protein(s) | #Pep | #Pep | #Pep | #Pep | #Clu | #Pep | #Clu | #Pep |
| c10861_g1_i1.p1 | 299 | 16 | 1275 | 1590 | 10 | 7204 |  |  |
| c10861_g1_i1.p1, c7216_g1_i1.p1 |  |  |  |  |  |  | 1 | 1174 |
| c13533_g1_i1.p1 |  | 48 | 516 | 564 | 3 | 2178 | 3 | 2819 |
| c13533_g1_i1.p1, c24_g1_i1.p2 |  |  |  |  | 1 | 1125 |  |  |
| c13559_g1_i1.p1, c6825_g1_i1.p3 | 66 |  | 12 | 78 | 2 | 1978 | 2 | 2944 |
| c13559_g1_i1.p1 | 172 |  | 204 | 376 | 1 | 923 |  |  |
| c14987_g1_i1.p1 | 465 | 33 | 1140 | 1638 | 12 | 8930 | 2 | 574 |
| c1545_g1_i1.p5 | 336 |  | 323 | 659 | 5 | 2346 |  |  |
| c17933_g1_i1.p1 | 1 |  | 712 | 713 | 5 | 3280 | 3 | 2739 |
| c24_g1_i1.p2 | 38 |  | 830 | 868 | 7 | 5837 | 4 | 3501 |
| c24_g1_i1.p2, c7502_g1_i1.p1 |  |  |  |  |  |  | 1 | 557 |
| c24_g1_i1.p2, c4419_g1_i1.p2 |  |  |  |  |  |  | 1 | 910 |
| c4419_g1_i1.p2 | 13 | 36 | 77 | 126 | 4 | 3096 | 2 | 1540 |
| c6405_g1_i2.p4 | 14 |  | 177 | 191 | 5 | 2363 | 3 | 3220 |
| c6656_g1_i1.p1 | 239 | 11 | 666 | 916 | 16 | 9797 | 3 | 3088 |
| c6825_g1_i1.p3 | 128 |  | 256 | 384 | 2 | 1840 |  |  |
| c6963_g1_i1.p1 | 92 | 10 | 468 | 570 | 7 | 3640 | 1 | 673 |
| c7216_g1_i1.p1 | 26 | 233 | 367 | 626 | 8 | 5841 | 4 | 3430 |
| c7502_g1_i1.p1 | 209 |  | 602 | 811 | 4 | 2953 | 2 | 1528 |
| c907_g1_i1.p2 | 2 | 147 | 414 | 563 | 4 | 2905 | 3 | 2789 |
| More than two parent proteins |  |  |  |  | 1 | 1082 | 5 | 7507 |
| Sum | 2100 | 534 | 8039 | 10673 | 97 | 67318 | 40 | 38993 |

For emulsifier peptides, α refers to peptides with predicted amphiphilic helical conformation, β refers to predicted amphiphilic sheet conformation, and γ refers to peptides with one end being mostly hydrophobic and the other mostly hydrophilic. For antioxidant peptides, SCA refers to predicted free radical scavengers while CHE refers to predicted metal chelators. #Pep refers to the number of individual peptides predicted from individual protein(s). For antioxidant peptides, #Clu refers to the number of predicted clusters and each cluster is assigned to the protein(s) representing the highest scoring peptide within each cluster.

**References**

Almagro Armenteros, J. J., Sønderby, C. K., Sønderby, S. K., Nielsen, H., & Winther, O. (2017). DeepLoc: prediction of protein subcellular localization using deep learning. *Bioinformatics*, *33*(21), 3387–3395. https://doi.org/10.1093/bioinformatics/btx431

García-Moreno, P. J., Gregersen, S., Nedamani, E. R., Olsen, T. H., Marcatili, P., Overgaard, M. T., … Jacobsen, C. (2020). Identification of emulsifier potato peptides by bioinformatics: application to omega-3 delivery emulsions and release from potato industry side streams. *Scientific Reports*, *10*(1), 690. https://doi.org/10.1038/s41598-019-57229-6

García-Moreno, P. J., Jacobsen, C., Marcatili, P., Gregersen, S., Overgaard, M. T., Andersen, M. L., … Hansen, E. B. (2020). Emulsifying peptides from potato protein predicted by bioinformatics: Stabilization of fish oil-in-water emulsions. *Food Hydrocolloids*, *101*, 105529. https://doi.org/10.1016/j.foodhyd.2019.105529

Gregersen, S., Pertseva, M., Marcatili, P., Holdt, S. L., Jacobsen, C., Garcia-Moreno, P. J., … Overgaard, M. T. (2020). Proteomic characterization of pilot scale hot-water extracts from the industrial carrageenan red seaweed Eucheuma denticulatum. *BioRxiv*, 2020.12.14.422673. https://doi.org/10.1101/2020.12.14.422673

Naseri, A., Holdt, S. L., & Jacobsen, C. (2019). Biochemical and Nutritional Composition of Industrial Red Seaweed Used in Carrageenan Production. *Journal of Aquatic Food Product Technology*, *28*(9), 967–973. https://doi.org/10.1080/10498850.2019.1664693

Naseri, A., Jacobsen, C., Sejberg, J. J. P., Pedersen, T. E., Larsen, J., Hansen, K. M., & Holdt, S. L. (2020). Multi-Extraction and Quality of Protein and Carrageenan from Commercial Spinosum (Eucheuma denticulatum). *Foods*, *9*(8), 1072. https://doi.org/10.3390/foods9081072

Olsen, T. H., Yesiltas, B., Marin, F. I., Pertseva, M., García-Moreno, P. J., Gregersen, S., … Marcatili, P. (2020). AnOxPePred: using deep learning for the prediction of antioxidative properties of peptides. *Scientific Reports*, *10*(1), 21471. https://doi.org/10.1038/s41598-020-78319-w

WHO/FAO/UNU. (2007). *PROTEIN AND AMINO ACID REQUIREMENTS IN HUMAN NUTRITION*. Retrieved from https://apps.who.int/iris/bitstream/handle/10665/43411/WHO_TRS_935_eng.pdf?sequence=1&isAllowed=y
